# Tuning the balance between dispersion and entropy to design temperature-responsive flexible metal-organic frameworks

**DOI:** 10.1038/s41467-018-07298-4

**Published:** 2018-11-21

**Authors:** J. Wieme, K. Lejaeghere, G. Kresse, V. Van Speybroeck

**Affiliations:** 1 Center for Molecular Modeling, Ghent University, Technologiepark 903, 9052 Zwijnaarde, Belgium; 2 Faculty of Physics and Center for Computational Materials Science, University of Vienna, Sensengasse 8/12, 1090 Vienna, Austria

## Abstract

Temperature-responsive flexibility in metal-organic frameworks (MOFs) appeals to the imagination. The ability to transform upon thermal stimuli while retaining a given crystalline topology is desired for specialized sensors and actuators. However, rational design of such shape-memory nanopores is hampered by a lack of knowledge on the nanoscopic interactions governing the observed behavior. Using the prototypical MIL-53(Al) as a starting point, we show that the phase transformation between a narrow-pore and large-pore phase is determined by a delicate balance between dispersion stabilization at low temperatures and entropic effects at higher ones. We present an accurate theoretical framework that allows designing breathing thermo-responsive MOFs, based on many-electron data for the dispersion interactions and density-functional theory entropy contributions. Within an isoreticular series of materials, MIL-53(Al), MIL-53(Al)-FA, DUT-4, DUT-5 and MIL-53(Ga), only MIL-53(Al) and MIL-53(Ga) are proven to switch phases within a realistic temperature range.

## Abstract (web summary)

Rational design of metal organic frameworks (MOFs) with shape-memory nanopores is a formidable challenge. Here the authors use an accurate theoretical approach to design thermo-responsive MOFs based on a balance of van der Waals and entropy contributions.

## Introduction

**Fig. 1:**
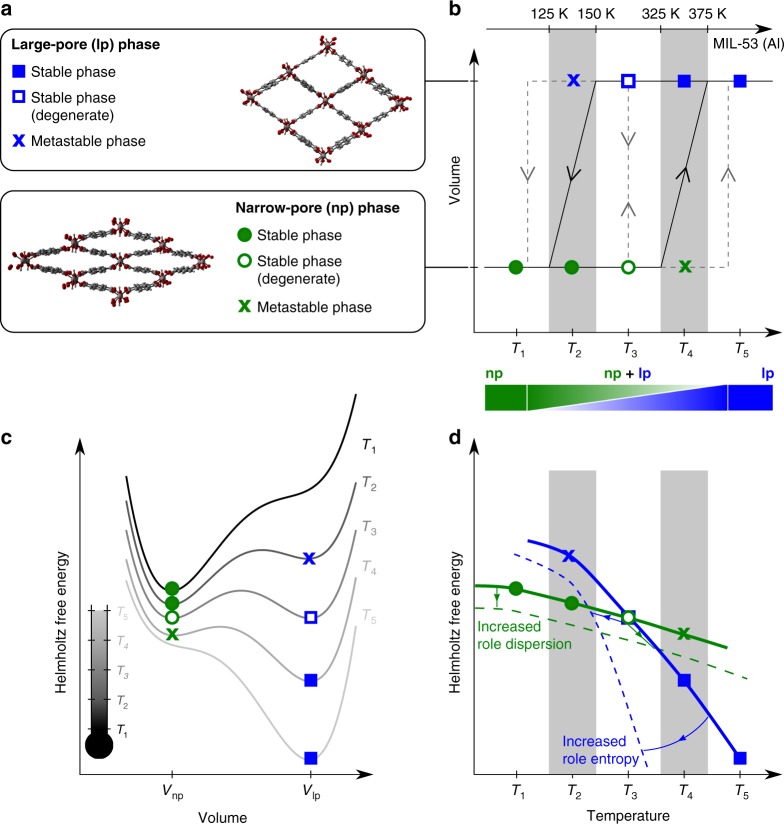
Temperature-induced breathing of a flexible MOF. The definition of the blue and green symbols is indicated in the top left panel. **a** Two (meta)stable phases of MIL-53(Al). **b** Thermo-responsive behavior of MIL-53(Al) as measured by experiment. The system switches as a function of temperature between the np and lp phase with a hysteresis loop. Different transition regimes are indicated with arrows (see main text). The gray shaded areas correspond to the experimentally measured transition regions^7^. **c** Hypothetical Helmholtz free energy curves as a function of volume at different temperatures (*T*_1_, *T*_2_, *T*_3_, *T*_4_, *T*_5_) as an explanation for the observed experimental behavior. **d** Hypothetical evolution of the Helmholtz free energy for the np and lp phase as a function of temperature. The gray shaded areas correspond to the experimentally measured transition regions^7^

The recent discovery of flexible porous materials ushered in an intriguing new era in science^1–4^. Their combination of cooperative structural transformability and crystallinity was initially perceived as counterintuitive. For some metal-organic frameworks (MOFs)^5,6^, such as the wine-rack-topology MIL-53 materials, phase transformations were observed between a narrow-pore (np) and large-pore (lp) phase (Fig. 1a)^7,8^. Such a phase transformation—also called breathing—is typically accompanied by large volume changes of about 40% and may be induced by external stimuli such as temperature, mechanical pressure, and adsorption of guest molecules^7,9–12^. These features are promising for applications such as controlled drug release^13^, gas adsorption and separation^10,14,15^, and sensors^16^. A particularly captivating case is that of temperature-responsive breathing. The search for a new generation of temperature-controlled smart materials attracts much interest^17–19^, in which thermally triggered breathing MOFs may play a pivotal role. Unfortunately, MOFs exhibiting this flexibility are rare^20–26^. Indeed, rational design of MOFs with shape-memory nanopores remains a formidable challenge^27,28^. Nowadays their discovery is based on serendipity, since the nanoscopic interactions and transition mechanisms governing the flexible behavior are not properly understood. Motivated by this lack of understanding, we investigate in this contribution the intrinsic physical origin of temperature-responsive breathing using thermodynamic considerations and state-of-the-art electronic-structure calculations.

Experimentally, framework flexibility can be followed by monitoring the response of a material as a function of an applied external stimulus. A breathing transition yields a sudden volume change at a given value of the external trigger. The principle is illustrated in Fig. 1 for MIL-53(Al), a material for which Liu et al.^7^ observed a reversible phase transformation as a function of temperature. A significant temperature hysteresis was found (Fig. 1b): the transition from the lp to the np structure occurs in a temperature window around *T*_2_, while the transition from the np to the lp structure takes place around *T*_4_. For MIL-53(Al), the experimental transition temperatures lie between 125–150 K and between 325–375 K, respectively. The experiments enable to unambiguously detect stimulus-responsive behavior, but they do not allow the construction of the underlying thermodynamic potential^4^ governing the observed behavior, like the Helmholtz free energy *F*. The experimental observations do, however, suggest that low-temperature Helmholtz free energies favor the np phase, whereas at higher temperature the lp phase prevails. Apart from that the observations provide little guidance for the development of new thermo-responsive materials.

A thorough understanding of the thermodynamic potential is critical for design of dedicated applications, e.g., tuning the transition temperatures for sensors and actuators. Some hypothetical Helmholtz free energy profiles in terms of the volume are shown in Fig. 1c for various temperatures. The low- (*T*_1_) and high-temperature (*T*_5_) profiles are extreme cases and a broad temperature window with bistable behavior is expected (*T*_2_–*T*_4_), as suggested by the observed hysteresis. The relative Helmholtz free energy difference between the two phases determines the possibility of a phase transformation as a function of temperature. Based on the hypothetical curves in Fig. 1c, the temperature dependence of the Helmholtz free energy of both phases (*F*_np_ and *F*_lp_) is sketched in Fig. 1d. To obtain an np-to-lp transition at *T*_4_ and an lp-to-np transition at *T*_2_, *F*_np_ needs to be lower than *F*_lp_ for temperatures below *T*_2_ and *F*_lp_ needs to be lower than *F*_np_ for temperatures higher than *T*_4_. Such behavior requires a critical crossing temperature of the two curves (*T*_cross_) at *T*_3_, i.e., between *T*_2_ and *T*_4_.

Theoretical investigations are indispensable to pinpoint the microscopic factors controlling the shape of the thermodynamic potential. Moreover, theory can overcome some puzzling dependences on the experimental synthesis procedure. Some groups found non-breathing MIL-53(Al) frameworks when procedures other than standard hydrothermal synthesis were used, for example^29–33^. These experiments indicate that the material could be trapped in a metastable phase due to interactions with the solvent^34^. Theoretical calculations are not hampered by such a critical experimental sensitivity and may therefore advance understanding and development of flexible MOFs. Ideally, theory can even predict materials properties of yet to be synthesized MOFs and stimulate the discovery of new flexible MOFs. Various groups explored computer-aided design by screening hypothetical MOFs for several desired properties and functions^35–38^.

Previous theoretical studies have suggested that the lp-np relative stability of MIL-53(Al) is dependent on two factors: long-range dispersion interactions and vibrational entropy^39,40^. Dispersion is related to the attractive part of a van der Waals-type nonbonding interaction, while vibrational entropy connects to the amount of freedom the atoms have to move. Walker et al.^40^ showed using density-functional theory (DFT) that long-range dispersion is critical to obtain the np phase at low temperatures, as it is mainly stabilized by π−π stacking interactions between the organic linkers. The strength of the dispersion interactions is, however, very method dependent (Supplementary Table 4); stronger dispersion interactions shift the *F*_np_ curve down with respect to *F*_lp_, yielding a higher *T*_cross_ (Fig. 1d). To date, there is no clear understanding on the impact of the chosen dispersion method on the observed thermo-responsive behavior. The stabilization of the lp phase at higher temperatures is caused by entropy^41^, as the linkers have more freedom to move in this phase. An increase of the entropic part of the Helmholtz free energy stabilizes the lp phase, yielding lower critical temperatures for the transition.

Herein, we critically assess the impact of dispersion interactions and thermal effects on the breathing behavior of MIL-53(Al) using state-of-the-art theoretical calculations. To ensure an accurate account of the 0 K lp-np relative stability, we present the first many-electron treatment of a flexible MOF by applying the random-phase approximation to the correlation energy (RPA). Our results provide unprecedented insight into the mechanism behind temperature-induced breathing, which enables formulating some minimal design rules for tailoring flexible MOFs with a temperature switch in a desired window. We demonstrate for a series of isoreticular materials that rational design of flexible MOFs is now within reach. In addition, our high-level computational approach shows some deficiencies in commonly used electronic-structure methods which may stimulate the theoretical community to investigate hybrid materials like flexible MOFs as a novel test bed for future theory development.

## Results

### Temperature-dependent stability of the np and lp phases

We calculated the harmonic Helmholtz free energy difference between np and lp MIL-53(Al) as a function of temperature using various exchange-correlation (XC) functionals and dispersion schemes. Table 1 lists the differences at 130 and 350 K, which are situated in the experimental transition windows^7^. None of the methods reproduce the expected relative stability for both phases. Because the np phase remains the most stable structure at both temperatures, the expected crossing point (Fig. 1d) is situated at too high temperatures (410–1474 K). As mentioned above, the relative position of the Helmholtz free energy curves of both phases is expected to be dictated by a delicate balance between dispersion and entropy. The individual contributions to the energy difference are given in Table 1 and show that the electronic energy is the dominating term for most methods in the experimental temperature window. There is a substantial theoretical spread on this energy (30 kJ mol^−1^), which confirms the strongly scattered DFT data in literature (Supplementary Table 4). The entropic contribution to the Helmholtz free energy at 300 K varies to a lesser extent (7 kJ mol^−1^). Getting the electronic energy difference right is therefore essential to accurately determine the overall position of *T*_cross_.

**Table 1:** Helmholtz free energy, energy and entropy difference between the lp and np phase of MIL-53(Al) for different methods

	Δ*F*_lp-np_ at 130 K (kJ mol^−1^)	Δ*F*_lp-np_ at 350 K (kJ mol^−1^)	*T*_cross_ (K)	Δ*E*^el^_lp-np_ (kJ mol^−1^)	Δ*E*^vib^_lp-np_ at 240 K (kJ mol^−1^)	−*T*Δ*S*^vib^_lp-np_ at 240 K (kJ mol^−1^)
PBE + D2	10.9	2.4	410	15.7	0.5	−9.4
PBE + MBD	20.8	13.3	735	24.5	0.3	−8.3
SCAN + rVV10	25.7	14.0	799	28.3	0.3	−10.3
vdW-DF2	31.2	21.0	799	35.5	2.6	−11.3
PBE + MBD/FI	15.0	11.3	1034	16.4	0.8	−4.2
PBE + D3(BJ)	24.1	19.1	1201	26.1	0.4	−5.5
PBEsol + D3(BJ)	41.4	34.6	1474	44.2	1.3	−7.5

The Helmholtz free energy difference Δ*F*_lp-np_ was calculated via the harmonic approximation for different DFT methods in the np-to-lp (at 130 K) and lp-to-np (at 350 K) transition range. The crossing temperature *T*_cross_, i.e., when both phases are in thermodynamic equilibrium (Δ*F*_lp-np_ = 0), is also given. The different contributions to the Helmholtz free energy are given in the last three columns: the electronic energy difference $\Delta E_{{\mathrm{lp}} - {\mathrm{np}}}^{{\mathrm{el}}}$, the vibrational energy difference $\Delta E_{{\mathrm{lp}} - {\mathrm{np}}}^{{\mathrm{vib}}}$ and the entropic contribution $- T\Delta S_{{\mathrm{lp}} - {\mathrm{np}}}^{{\mathrm{vib}}}$. The latter two depend on temperature and are given for a value in the middle of the experimental hysteresis loop

**Fig. 2:**
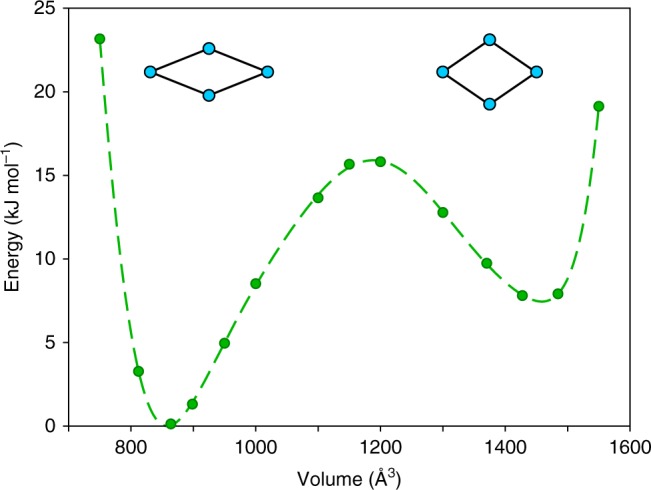
RPA + SE electronic energy profile as a function of volume for MIL-53(Al)

This knowledge motivated us to construct high-accuracy data for the electronic energies using the random-phase approximation. This many-body perturbative approach to the correlation energy goes beyond the state of the art with respect to the computational treatment of flexible porous materials. It seamlessly integrates long-range dispersion effects with short-range interactions. Although beyond-RPA contributions may improve the performance even further (see Methods section), it already results in an excellent agreement with experimental data for various systems^42–45^. Because of the importance of single-excitation (SE) effects for molecular solids^46–48^, this contribution was moreover included and found to be crucial for our system as well (Supplementary Figure 5 in Supplementary Note 7). The energy-versus-volume profile of MIL-53(Al) at the RPA + SE level of theory is shown in Fig. 2. The energy difference between the lp and np phase amounts to only 7.4 kJ mol^−1^. Furthermore, a barrier of more than 8 kJ mol^−1^ is found in both directions, facilitating the existence of a metastable lp state even at 0 K.

**Fig. 3:**
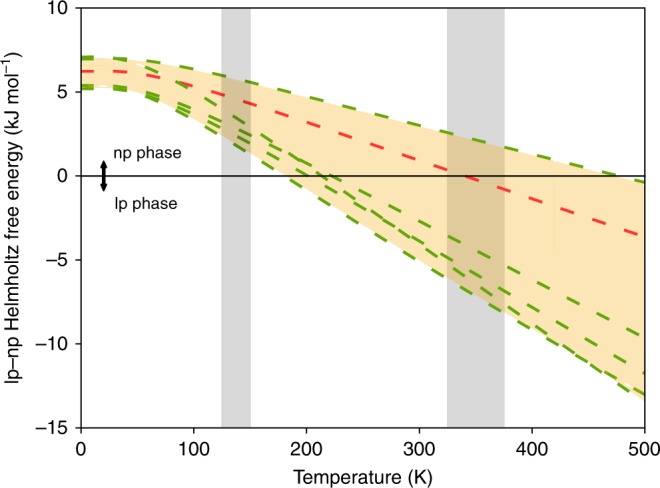
Helmholtz free energy difference between the lp and np phase of MIL-53(Al) as a function of temperature. This Helmholtz free energy difference was calculated with the harmonic approximation. An accurate electronic energy difference between the lp and np phase was obtained using RPA + SE. The displayed spread (orange shaded area) is obtained using predictions of the evolution of the Helmholtz free energy difference with various density-functional theory methods (Table 1). The red curve indicates the corrections at the PBE + D3(BJ) level of theory, while the green curves represent the other levels of theory (from bottom to top starting from the right: vdW-DF2, SCAN + rVV10, PBE + D2, PBE + MBD, PBEsol + D3(BJ) and PBE + MBD/FI). The individual Helmholtz free energy curves are shown in more detail in Supplementary Note 6 (Supplementary Figure 4). The gray shaded areas correspond with the experimentally measured transition regions^7^

Using these high-accuracy data for the electronic energies, we reassessed the position of *T*_cross_. Because current computational resources do not yet allow to calculate RPA + SE thermal corrections for large systems like MOFs, Fig. 3 shows Δ*F*_lp-np_(T) with thermal corrections at various DFT levels (Table 1) on top of the RPA + SE electronic energy difference. It was previously shown that the specific choice of the XC functional and dispersion scheme could influence the thermal corrections and relative stability at room temperature in, for example, molecular crystals^49^. The orange region in Fig. 3 delimits the method dependence of the zero-point, vibrational and entropic contributions. Starting from the RPA + SE-favored np at 0 K, most methods change sign in the experimentally determined temperature window between *T*_2_ and *T*_4_. Combining an accurate electronic energy difference with standard DFT vibrational entropy contributions hence yields a crossing temperature in accordance with experiment^7,50^ and thermodynamic models^51,52^.

As RPA + SE is computationally too demanding to be employed on a scale required for materials design, it is important to identify cheaper DFT methods with a similar predictive quality^53^. We therefore compared our DFT data to the RPA + SE benchmark and extended the set of assessed DFT methods to more advanced XC and dispersion schemes. Table 2 confirms that despite the omnipresent use of DFT in materials science, flexible MOFs are a challenging case for common DFT-based methods, which should be used with caution. Nonetheless, several interesting trends can be extracted. While dispersion corrections are necessary to stabilize the np phase, most methods overbind it, some even to the extent that they fail to find a metastable lp state. Although a rather large spread is observed on the equilibrium volumes too, we show in Supplementary Note 3 (Supplementary Figures 2 and 3, Supplementary Tables 7–15) that the structural differences affect the lp-np energy difference only to a limited extent. The deviations are mostly due to the used dispersion model. Many popular methods, such as the Grimme D2 and D3 schemes, describe dispersion by means of purely pairwise atomic interactions. However, higher-order corrections to the dispersion interaction were reported to play an important role in some other materials^54–57^, and are now found to be crucial to get the relative phase stability of MIL-53(Al) right. Indeed, many-body effects (MBD vs TS and D3(BJ)^ATM^ vs. D3(BJ)) destabilize the np phase and bring relative energy differences much closer to the benchmark value. This is also reflected in the equilibrium volumes of both phases, which are in general too small, but improve for schemes beyond pairwise atomic interactions. We further note that a recent adaptation of the promising MBD scheme, MBD/FI, performs better than the original. This can probably be ascribed to the revised description of the polarizability for systems with a strongly ionic character, relevant for the one-dimensional metal-oxide chain in MIL-53(Al).

**Table 2:** The electronic energy difference between the lp and np phase of MIL-53(Al) with different methods

	*V*_np_ (Å^3^)	*V*_lp_ (Å^3^)	Δ*E*^el^_lp-np_ (kJ mol^−1^)	Δ*E*^el,*^_lp-np_ (kJ mol^−1^)
Experiment (77 K)^7^	864	1419^a^	–	–
RPA + SE	860	1455	7.4	7.7 ± 3.5^b^
PBE	np unstable	1489	np unstable	-118.5
PBE + D2	808	1457	15.7	12.8
PBE + D3(BJ)	843	1426	26.6	26.1
PBE + D3(BJ)^ATM^	874	1448	5.8	6.2
HSE06 + D3(BJ)	825	lp unstable	lp unstable	42.3
HSE06 + D3(BJ)^ATM^	850	1389	22.3	22.4
B3LYP + D3(BJ)	789	lp unstable	lp unstable	58.9
B3LYP + D3(BJ)^ATM^	806	1405	43.8	39.0
PBE + TS	793	lp unstable	lp unstable	74.2
PBE + MBD	828	1425	25.8	24.5
PBE + MBD/FI	851	1439	16.4	16.4
SCAN	np unstable	1430	np unstable	-43.6
SCAN + rVV10	803	1384	31.2	28.3
vdW-DF2	869	1456	34.8	35.5
M06-L	829	lp unstable	lp unstable	36.6

$\Delta E_{{\mathrm{lp}} - {\mathrm{np}}}^{{\mathrm{el}}}$ is the 0 K electronic energy difference at the equilibrium volumes of both phases (*V*_np_ and *V*_lp_) relaxed with the specific method (or PBE + D3(BJ) for RPA + SE, see Methods). $\Delta E_{{\mathrm{lp}} - {\mathrm{np}}}^{{\mathrm{el}}, \ast }$ is the 0 K electronic energy difference at fixed structures (864 Å^3^ (np) and 1427 Å^3^ (lp)). The lp and np structures are provided in Supplementary Data 1 and 2. Generalized gradient approximation (GGA), meta-GGA and hybrid XC functionals were included together with dispersion models ranging from pairwise (D2, D3(BJ), TS) to many-body schemes (D3(BJ)^ATM^, MBD, MBD/FI). Three XC functionals inherently including long-range dispersion were also tested, i.e., SCAN + rVV10, vdW-DF2 and M06-L. A full list of references to these methods is included in the Methods section

^a^The sample was going towards the np phase on a longer time scale than the measurement^7^

^b^The error bar on the RPA + SE result reflects the uncertainty due to numerical convergence effects. More information on how this error bar was determined can be found in the Methods section

Overall PBE + D3(BJ)^ATM^ yields an energy difference of 6.2 kJ mol^−1^ in closest agreement with the reference RPA + SE value for this particular system. One should nevertheless be cautious to extrapolate the good performance of this dispersion method to other materials. A key problem lies in the choice of the dispersion model parameters and the coupling with the XC functional. For van der Waals-type bonding, dispersion corrections are designed to yield results largely independent of the parent functional for a set of reference molecules. Even though MOFs are not included in this set, after adding dispersion corrections, the energy difference between the lp and np becomes reasonably independent on the parent functional. This compensation is indeed observed for TS and MBD, but it is less effective for D3-derived methods (Supplementary Tables 16 and 17). This might explain why the success of PBE + D3(BJ)^ATM^ does not transfer to HSE06 + D3(BJ)^ATM^. In addition the SCAN functional yields results that are less satisfactory than one might hope^58,59^. As we show in Supplementary Note 4 (Supplementary Table 16), this effect is related to the nontrivial coupling between the SCAN functional and dispersion schemes^60^. A meticulous choice of both the XC functional and the dispersion scheme is therefore of utmost importance to obtain reliable energy values for flexible MOFs, which are prone to subtle long-range interactions. The above discussion underlines the complex physics in MIL-53(Al) and the challenge of correctly capturing it. The PBE + D3(BJ)^ATM^ method serves as a convenient and cheap solution for the system at hand, although this might be somewhat accidentally.

### Transition mechanism and barrier between np and lp

The approach used so far identifies the thermodynamically preferred phase at various temperatures. Indeed, bistable behavior is a necessary condition for breathing. However, bistability alone is not sufficient to guarantee temperature-responsive flexibility. The transition mechanism determines whether it is possible to reversibly switch between the two phases. Two limiting scenarios may be distinguished, as schematically shown in Fig. 1b. If the stability of the two phases were the only criterion, both the forward and backward transition would occur at the temperature *T*_3_ where the two phases become isoenergetic, and no hysteresis would be found. In the other extreme case, a phase transition would only take place when the barrier between the two (meta)stable states fully disappears and only one minimum is present in the Helmholtz free energy curve. This mechanism corresponds to the hypothesis of collective behavior, where the entire framework must transform in a collective fashion. Even the smallest barrier in the thermodynamic potential of a single unit cell then translates to a huge barrier for the entire system^61^. Under this assumption the transition would happen between *T*_4_ and *T*_5_ for np to lp, and between *T*_2_ and *T*_1_ for lp to np (Fig. 1d).

Our high-accuracy RPA + SE data in Figs. 2 and 3 suggest an intermediate transition scenario. The experimentally observed hysteresis confirms the importance of the barrier between lp and np, but the RPA + SE barrier is too high to completely disappear within a reasonable temperature range. Fully collective behavior is therefore unrealistic. This conclusion is corroborated by Mendt et al., who found a fraction of lp material at temperatures down to 9 K^50^. Instead, the transition may be facilitated by surfaces or lattice imperfections, which serve as nucleation sites. Recent experimental studies showed that guest-responsive breathing of several MOFs is critically affected by the number of defects^27,62,63^. Larger crystals, which contain more defects, kinetically promote the phase transition yet maintain the center of the hysteresis loop^27^. Triguero et al. proposed a theoretical layer-by-layer transition model, which assumes that a collective lp-np transformation only occurs in a single layer of MIL-53(Al) at a time^64^. This idea is supported by first-principles molecular dynamics of guest-induced breathing in MIL-53(Sc)^65^. The temperature-induced transition of a framework may therefore nucleate through a combination of defects, temperature fluctuations and strains, after which the locally increased stress causes the transformation to propagate further through the lattice in a layer-by-layer fashion. In view of this suggested complex transition mechanism, the RPA + SE barrier should be considered as one of several parameters determining the experimental transition temperature.

### Design of temperature-induced flexibility

Our analysis of MIL-53(Al) identified the mechanisms underlying temperature-dependent breathing, putting particular emphasis on the shift from long-range dispersion stabilization at low temperature to entropic stabilization at higher temperatures. We can use this knowledge to rationalize changes in the crossing temperature for materials isoreticular to MIL-53(Al). Inspired by the good correspondence with RPA + SE for MIL-53(Al), we consider a series of materials with very similar structural characteristics and apply PBE + D3(BJ)^ATM^ for the electronic energy difference in combination with thermal corrections at the PBE + D3(BJ) level. Note that if flexible MOFs with a more different make-up would be considered, the most appropriate levels of theory for this procedure may vary and need to be rechecked. As an initial design strategy, we modulate the framework by changing the organic linkers. MIL-53(Al)-FA^66^, DUT-4^67,68^, and DUT-5^67^ have a similar inorganic chain as MIL-53(Al) but are connected with fumaric acid, biphenyl and naphthalene dicarboxylate linkers, respectively (see also Supplementary Table 19 in Supplementary Note 8).

**Fig. 4:**
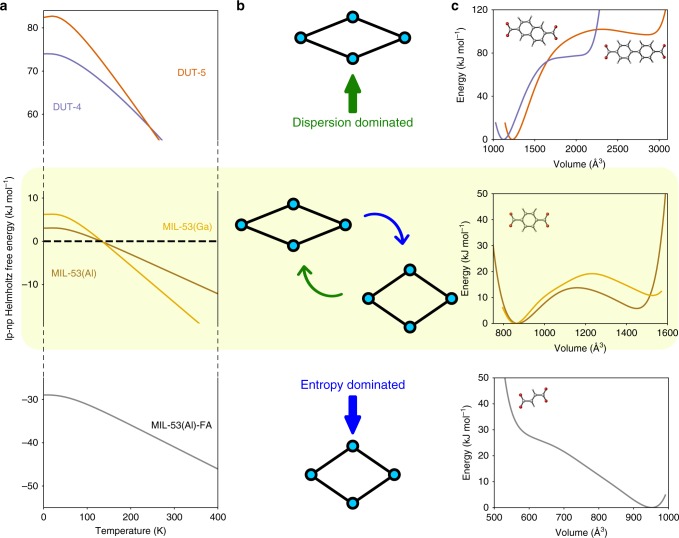
Design of temperature-dependent flexibility in isoreticular MIL-53 materials. Several experimentally synthesized materials are considered: MIL-53(Al)-FA (or A520)^66^, MIL-53(Al)^8^, MIL-53(Ga)^69^ (or IM-19^70^), DUT-4^67^ (or MIL-69^68^), and DUT-5^67^. The yellow shaded area highlights the desired temperature-responsive case. **a** Helmholtz free energy difference between the lp and np phase as a function of temperature for the series of isoreticular MIL-53 frameworks. The harmonic temperature corrections were calculated at the PBE + D3(BJ) level of theory, and were added on top of the 0 K PBE + D3(BJ)^ATM^ electronic energy difference. **b** Schematic depiction of the (meta)stable phases and the dominating contributions. **c** Electronic energy profiles as a function of the volume for different materials at the PBE + D3(BJ)^ATM^ level of theory. The corresponding organic linkers are indicated in the different panels

Figure 4 shows the electronic energy profiles at 0 K (Fig. 4c) and the Helmholtz free energy differences between the lp and np phase as a function of temperature (Fig. 4a). The lack of π−π stacking interactions limits the dispersion stabilization of the np phase in MIL-53(Al)-FA, while on the contrary, the extended linkers in the case of DUT-4 and DUT-5 stabilize it excessively. The Helmholtz free energy moreover varies more rapidly for DUT-5 as a function of temperature due to the mobility of the biphenyl linker in the lp phase. The easy rotation of the phenyl rings relative to each other gives rise to an increased lp-np entropy difference compared to the rigid naphthalene linker in DUT-4, for example. However, none of the considered frameworks is expected to display temperature-induced breathing. MIL-53(Al)-FA assumes an lp structure over the entire temperature range, and the stability of the np phase of DUT-4 and DUT-5 will never be compensated by entropy effects at temperatures for which the material remains stable. Changing the organic linkers thus results in a large shift of the Helmholtz free energy difference, which leaves little room for fine-tuning the temperature window for applications in these types of frameworks.

To achieve a subtler effect, we modified the inorganic chain by replacing aluminum with gallium^69,70^. As the coordination environment around gallium is more diffuse, it can deform more easily. This in turn enables the framework to reduce the strain energy penalty in the distorted dispersion-dominated dense phase, yielding an overall np stabilization. On the other hand, the entropy of the lp phase is also raised with respect to the np phase, overall compensating the effect on *T*_cross_ for MIL-53(Ga) and resulting in an only negligible shift (Fig. 4a).

Our findings for this series of materials are validated by the limited available experimental literature (see also Supplementary Table S20). MIL-53(Al)-FA resides in the lp phase at room temperature, and was only reported to reversibly breathe under influence of pressure^71,72^. DUT-4 has been hydrothermally synthesized in an np phase—also called MIL-69^68^—and remains nonporous up to more than 600 K, after which the structure decomposes. Note that although DUT-4 and DUT-5 could also be synthesized in an lp phase using DMF^67^, similar to MIL-53(Al)^30^, they were trapped in this metastable state. They do not show intrinsic flexibility as, for instance, lp DUT-4 could not be converted to its stable nonporous state. Finally, MIL-53(Ga) was experimentally found to be in a nonporous np phase at room temperature and undergoes a phase transition to the lp phase between 450 and 520 K^69,70,73^. This is consistent with our crossing temperature, even if a quantitative prediction of the transition range would require more insight in the transition mechanism, as discussed above.

## Discussion

As evidenced by our study of MIL-53-type MOFs, temperature-induced breathing can only be achieved when a delicate balance between several constraints is fulfilled. It suggests why thus far only a limited number of materials were found with this feature. To design a material with temperature-induced breathing behavior, at least two (meta)stable phases should exist, which are required to change relative stability as a function of temperature. The np phase should be stabilized at low temperatures by supramolecular interactions, while entropic contributions to the Helmholtz free energy drive the relative stability towards the lp phase with increasing temperature. The stability switch needs to occur at temperatures for which the material is still thermally stable. The exact transition temperature is dependent on the transition mechanism but the change of sign of the lp-np Helmholtz free energy difference is a necessary condition to obtain a temperature-dependent phase switch. These clear design rules could stimulate experimentalists to develop the next-generation of thermo-responsive MOFs.

A theoretical assessment of the above constraints only becomes possible when a sufficiently accurate level of theory is used. DFT calculations may be ubiquitous in materials science, but for flexible MOFs most common DFT approaches fall short. The XC functional and dispersion scheme need to be selected carefully as they largely determine the Helmholtz free energy difference for these materials. We took a major step forward by accurately determining the phase stability of the prototypical breathing material, MIL-53(Al), using computationally demanding high-level RPA + SE calculations. Comparison with various commonly used electronic-structure methods revealed some interesting physical trends, and exposed at the same time some issues with popular XC functionals and dispersion corrections. This will hopefully stimulate the community developing functionals to investigate the case of flexible MOFs carefully. Because the cheaper PBE + D3(BJ)^ATM^ method reasonably reproduced the benchmark results for MIL-53(Al), we were able to extend the discussion to a broader set of isoreticular materials, where we could possibly benefit from error compensation. However, care must be taken to extend this level of theory towards widely different materials as a sensible selection of the best functional is certainly system dependent. Future refinement of temperature effects may be obtained via more advanced estimates for the entropy, e.g., using molecular dynamics simulations^74^.

Temperature-induced breathing has the potential to define an entirely new class of temperature-triggered sensors and actuators, provided materials can be designed with a tunable switch in the desired temperature window. Np-to-lp phase transitions can be tuned by finding the right balance between dispersive and entropic effects. One option for modification exists in changing the organic linkers. The modified dispersion interaction hugely impacts the relative stability and thus the operation temperature window. Alternative and more subtle pathways include linker functionalization and local changes to the metal coordination. In these cases, shifts in electron density and changes in framework rigidity affect the np stabilization less directly. Additionally, entropic design may exploit linkers with larger mobility to enhance lp stability and decrease transition temperatures. The insights obtained here show that dispersive and entropic effects need to be tuned appropriately and pave the way to design thermo-responsive MOFs for desired applications.

## Methods

### General settings

All calculations of MIL-53(Al) were carried out with the Vienna Ab initio Simulation Package (VASP)^75^ using the projector-augmented wave (PAW) method^76,77^. The computational unit cell contains four organic linkers and thus 76 atoms in total. We employed the recommended GW-ready PBE PAW potentials for all elements and exchange-correlation (XC) functionals (v5.4). For the Al atoms, 3s and 3p electrons were explicitly included. For the C and O atoms, the 2s and 2p electrons were considered as valence electrons. For the H atoms, the 1s electron was explicitly treated. The specific settings for the density-functional theory (DFT) and random-phase approximation (RPA) simulations are listed below.

### DFT calculations

All DFT calculations on MIL-53(Al) were performed with a plane-wave kinetic-energy cutoff of 600 eV and using Gaussian smearing with a smearing width of 0.05 eV. Projection operators were evaluated in reciprocal space. A 2 × 6 × 6/2 × 6 × 2 Monkhorst-Pack k-grid was used for the np/lp phase^78^. The real-space Fast Fourier Transform (FFT) grid was able to describe wavevectors up to two times the maximum wavevector present in the basis set. An augmentation grid that is twice as large was used to avoid wrap-around errors in order to obtain accurate forces. The electronic (ionic) convergence criterion was set to 10^−8^ (10^−7^) eV, except for the hybrid XC functionals where it was slightly lowered to 10^−6^ (10^−5^) eV. The Hartree-Fock (HF) kernel for the latter was evaluated on a k-grid reduced by a factor of two in each reciprocal direction, which we found to be possible without a loss of accuracy^79^.

We considered GGA (generalized gradient approximation)^80–82^, meta-GGA^59,83,84^ and hybrid^85–87^ XC functionals, combined with dispersion schemes ranging from pairwise additive^88–91^ to many-body approaches^92–95^. In addition, we included XC functionals in which some long-range dispersion is inherently captured, such as vdW-DF2^80^, M06-L^84^, and SCAN + rVV10^59^. Most DFT simulations executed in this work consist of fixed-volume relaxations in which the positions and cell shape were optimized. The equilibrium volumes were obtained by constructing a local energy profile as a function of the volume, and by fitting a Rose-Vinet equation of state to the profile (Supplementary Figure 1)^78,96^. Only for the DFT-D3 schemes including Axilrod-Teller-Muto (ATM)^97,98^ contributions (DFT-D3^ATM^), optimizations were performed at the DFT-D3 level. ATM corrections were calculated with the DFT-D3 program and added a posteriori to the energy, as they have not yet been implemented in VASP. This is justified by the typically small corrections to the geometry^57^. An overview of the different methodologies used in this work is given in Supplementary Note 1 (Supplementary Tables 1-3). An overview of the available experimental information (Supplementary Note 2) and our DFT optimized structures (Supplementary Note 3) is given in Supplementary Tables 5, 6, 8 and 9.

Furthermore, we considered other materials isoreticular to MIL-53(Al). We mostly used the same computational settings for the DFT calculations on MIL-53(Al)-FA, MIL-53(Ga), DUT-4 and DUT-5. Only for the last two materials, the k-point grid of the np phase was lowered to a 1 × 6 × 6 mesh. The computational unit cell contains four linkers for all materials. We also employed the recommended GW-ready PAW potential for the Ga atoms, which includes the 3d, 4s, and 4p electrons explicitly.

We calculated the Helmholtz free energy using the harmonic approximation for some well-known methods predicting a bistable material. To this end, we determined the dynamical matrix using 0.01 Å displacements for all atomic coordinates in a 1 × 2 × 1 supercell. We only included Γ-point phonons, such that the expressions for the energy, entropy and the Helmholtz free energy in the harmonic approximation simplify to:

1 $${E}\left( T \right) 	= E^{{\mathrm{el}}} + {\sum \limits_{i = 1}^{3N}} \left( {\frac{{\hbar \omega _i}}{2} + \frac{{\hbar \omega _i}}{{e^{\frac{{\hbar \omega _i}}{{{k}_{\mathrm{B}}T}}} - 1}}} \right)\\ 	= E^{{\mathrm{el}}} + E^{{\mathrm{ZPE}}} + {\sum \limits_{i = 1}^{3N}} \frac{{\hbar \omega _i}}{{e^{\frac{{\hbar \omega _i}}{{k_{\mathrm{B}}T}}} - 1}}$$

2 $$S\left( T \right) = {k}_{\mathrm{B}}\mathop {\sum }\limits_{i = 1}^{3N} \left( {\frac{{\hbar \omega _i}}{{{k}_{\mathrm{B}}T}}\frac{1}{{e^{\frac{{\hbar \omega _i}}{{{{k}}_{\mathrm{B}}T}}} - 1}} - \ln \left( {1 - e^{ - \frac{{\hbar \omega _i}}{{{k}_{\mathrm{B}}T}}}} \right)} \right)$$

3 $$F\left( T \right) = E\left( T \right) - TS\left( T \right) = E^{el} + \mathop {\sum }\limits_{i = 1}^{3N} \left( {\frac{{\hbar \omega _i}}{2} +{{k}}_{\mathrm{B}}T\ln \left( {1 - e^{ - \frac{{\hbar \omega _i}}{{{{k}}_{\mathrm{B}}T}}}} \right)} \right)$$

The post-processing of the results was performed with phonopy^99^.

### RPA calculations

For the RPA calculations on MIL-53(Al), we applied a cubically scaling algorithm proposed and implemented in VASP by Kaltak et al.^100,101^ Such calculations combine the HF energy and RPA correlation energy using non-self-consistent calculations on top of DFT Kohn-Sham orbitals. These orbitals were generated using the SCAN meta-GGA functional^58,83^, which was proven to provide a good starting point for non-self-consistent RPA^102^. We moreover included single-excitation contributions by calculating the difference between the exact exchange energy calculated using frozen SCAN orbitals and the energy obtained by a single diagonalization of the Hartree-Fock Hamiltonian set up using the SCAN orbitals^102^. As shown in ^47^ this is practically equivalent to the singles of Ren and coworkers^48^.

All RPA calculations were performed with a plane-wave kinetic-energy cutoff of 600 eV and using Gaussian smearing with a smearing width of 0.05 eV. Both for the semilocal parts and the exchange energy (EXX), the real-space FFT grid was able to describe wavevectors up to 3/2 times the maximum wavevector present in the basis set and two times as many for the grid representing the augmentation charges. Projection operators were evaluated in reciprocal space. The RPA correlation energy was determined for a 2 × 3 × 3 (750–1150 Å^3^) or 2 × 3 × 2 Γ-centered k-grid (1200–1550 Å^3^), while higher k-point settings were needed for the EXX energy: 3 × 8 × 8 for the smaller cells and 3 × 8 × 5 for the larger cells. Singles contributions were calculated at 3 × 5 × 5 and 3 × 5 × 3 k-grid respectively. For the RPA correlation energies, the long-wavelength contribution was neglected, which is a good approximation in case of sufficiently dense k-grids^103^. In addition, the response function was expanded into plane waves up to several values of the wavevector, after which the correlation energy was extrapolated to the asymptotic limit of infinite cutoffs^103,104^. Integration of the response function over frequency space^100^ was performed using eight frequency points. For the contribution of single excitations, we needed to optimize wavefunctions, which were determined by means of a blocked Davidson iteration scheme using up to 10 iterations per band. Convergence was assumed as soon as the change in energy eigenvalue had decreased by a factor 1000.

Using the above settings, we estimate the numerical error bar on our RPA lp-np energy difference to be 3.5 kJ mol^−1^. Increasing the number of k-points for the MIL-53(Al) lp EXX energy from 3 × 8 × 5 to 4 × 10 × 6 yielded a difference of only 6 µeV, and going from 2 × 3 × 3 / 2 × 3 × 2 to 2 × 5 × 5 / 2 × 5 × 3 (with a cutoff of 400 eV) changed the RPA correlation energy difference between the lp and the np phase by 8 meV. Singles contributions were found to display a similar convergence behavior as the EXX energy, leading to an estimated 0.2 meV difference between 3 × 5 × 5 / 3 × 5 × 3 and 3 × 8 × 8 / 3 × 8 × 5 k-grids. Raising the cutoff energy did not significantly change the relative stability of the lp and np phase: going from 600 eV to 700 eV changed the EXX energy difference by 2 meV, the RPA energy difference by 0.5 meV and the singles energy difference by 0.01 eV. The largest influence was found to be the reference determinant for the non-self-consistent RPA calculations. Changing the starting orbitals from SCAN to PBE affects the lp-np energy difference by 0.03 eV and the singles contribution by 0.015 eV. The latter error bar also captures deviations due to using a Davidson optimization rather than an exact diagonalization. Differences due to this approach were not larger than 2 meV in the neighborhood of the np volume. When assuming all error contributions to be largely independent, we arrive at a total of 36 meV or 3.5 kJ mol^−1^ on the energy difference between the lp and np phase.

Note that the proposed error bar of 3.5 kJ mol^−1^ only captures numerical effects and does not consider the limitations of the RPA + SE theory itself. From the viewpoint of many-body perturbation theory, RPA only sums ring diagrams which describe the nonlocal coupling between quantum charge fluctuations. This gives rise to a systematic underbinding^105^. Several methods have therefore been proposed to include other diagrammatic contributions (e.g., ^106,107^). Specifically, different approaches exist to account for the effect of single excitations^47,48^. This may give rise to additional uncertainty. Unfortunately, it is difficult to estimate the magnitude of these errors for MIL-53(Al). Since DFT polarizabilities are typically within a few percent of experimental data and coupled-cluster results^108^, the leading RPA error is expected to stem from an overestimation of the Pauli repulsion due to the use of DFT orbitals for the exchange, which we captured by taking into account single excitations. A rough estimate of the residual error can be obtained by comparing our results to the hybrid approach proposed by Ren et al., which involves calculating the Hartree-Fock exchange fully self-consistently^48^. This results in a 10.8 kJ mol^−1^ lp-np energy difference, i.e., a 3.1 kJ mol^−1^ difference from our previously reported RPA + SE result. We therefore expect theory-based errors to be of the same order as the numerical errors. The limited size of the total error is also corroborated by the good agreement of the predicted lp-np crossing temperatures compared to experiment when an RPA + SE reference is used (see Table 1 and Fig. 3).

Although it has recently become possible to calculate RPA forces within the VASP PAW formalism^109^, structural relaxations of the MIL-53(Al) unit cell are still beyond the capabilities of current computing infrastructures. We have therefore performed single-point RPA + SE calculations on top of DFT-optimized crystal structures. We selected PBE + D3(BJ) for crystal structure generation (Supplementary Data 1 and 2) as this method not only yields good geometries, but also provides reasonable lp-np energy differences and volumes at an acceptable cost (Supplementary Data 1 and 2). We investigated the sensitivity of the RPA + SE lp-np energy difference by testing lp and np geometries from vdW-DF2 and HSE06+D3(BJ) and the influence on the relative stability was within the RPA + SE error bar (Supplementary Table 18 in Supplementary Note 5).

### Code availability

The VASP code can be licensed from the University of Vienna (see the FAQ section on https://www.vasp.at/).

## Electronic supplementary material

Supplementary Information

Description of Additional Supplementary Files

Supplementary Data 1

Supplementary Data 2

## Data Availability

The authors declare that all data supporting the findings of this study are available within the paper and the Supplementary Files, or available from the authors upon request.
