# Supplementary material for: Tuning the balance between dispersion and entropy to design temperature-responsive flexible metal-organic frameworks
Source: Nat Commun. 2018 Nov 21;9:4899. doi: 10.1038/s41467-018-07298-4 (PMC6249296; doi:10.1038/s41467-018-07298-4)
Supplement: Supplementary file 1 — Supplementary Information [file 41467_2018_7298_MOESM1_ESM.pdf]

Supplementary Information

Tuning the balance between dispersion and entropy to design temperature-responsive flexible metal-organic frameworks

Wieme et al.

Supplementary Note 1. DFT calculations

An overview of and the references to the different methodologies used in this work are given in Supplementary Tables 1, 2 and 3. An overview of previously published energy differences between the large-pore (lp) and narrow-pore (np) phase of MIL-53(Al) is given in Supplementary Table 4.

Name	*Abbreviation*	*Reference(s)*	*Type*
Perdew-Burke-Ernzerhof	PBE	[1, 2]	GGA
Perdew-Burke-Ernzerhof revised for solids	PBEsol	[3, 4]	GGA
van der Waals density functional 2	vdW-DF2	[5-8]	GGA
Strongly Constrained and Appropriately Normed	SCAN	[9, 10]	meta-GGA
Strongly Constrained and Appropriately Normed with revised Vydrov-Van Voorhis van der Waals correlation	SCAN+rVV10	[9-13]	meta-GGA
Minnesota ’06 (local)	M06-L	[14]	meta-GGA
Becke three-parameter Lee-Yang-Parr	B3LYP	[15-18]	hybrid
Heyd-Scuseria-Ernzerhof	HSE06	[19-21]	hybrid

Supplementary Table 1. Different exchange-correlation functionals used in this work.

Name	*Abbreviation*	*Reference(s)*
Grimme D3 dispersion with Becke-Johnson damping	D3(BJ)	[22, 23]
Grimme D3 dispersion with Becke-Johnson damping and Axilrod-Teller-Muto three-body potential	$D3(BJ)^{ATM}$	[22-24]
Grimme D2 dispersion	D2	[25]
Tkatchenko-Scheffler method	TS	[26]
Many-body dispersion energy method (MBD@rsSCS)	MBD	[27-30]
Many-body dispersion energy method with a fractionally ionic approach to polarizability (MBD@rsSCS/FI)	MBD/FI	[31]

Supplementary Table 2. Different dispersion schemes used in this work.

Name	*XC functional*	*Dispersion scheme*
PBE	PBE	-
PBE+D3(BJ)$^{(ATM)}$	PBE	D3(BJ)$^{(ATM)}$
PBE+TS	PBE	TS
PBEsol+D3(BJ)$^{(ATM)}$	PBEsol	D3(BJ)$^{(ATM)}$
PBE+D2	PBE	D2
PBE+MBD	PBE	MBD
PBE+MBD/FI	PBE	MBD/FI
SCAN	SCAN	-
SCAN+rVV10	SCAN+rVV10	-
vdW-DF2	vdW-DF2	-
M06-L	M06-L	-
B3LYP+D3(BJ)$^{(ATM)}$	B3LYP	D3(BJ)$^{(ATM)}$
HSE06+D3(BJ)$^{(ATM)}$	HSE06	D3(BJ)$^{(ATM)}$

Supplementary Table 3. Overview of DFT methods used in this work.

Reference	*XC functional*	*Dispersion scheme*	*Code*	$\Delta E_{\mathrm{lp-np}}$ (kJ·mol^{-1})
Walker *et al.* (2010) [32]	vdW-DF	-	SIESTA	72
Walker *et al.* (2010) [32]	PBE	D2	CRYSTAL09	39
Walker *et al.* (2010) [32]	B3LYP	D2	CRYSTAL09	42
Walker *et al.* (2010) [32]	B3LYP	D2*	CRYSTAL09	34
Nanthamathee *et al.* (2015) [33]	PBE	D3^{ATM}	CP2K	16
Ling and Slater (2015) [34]	HSE06	D3^{ATM}	CP2K	14
Coudert *et al.* (2014) [35]	B3LYP	D2	CRYSTAL09	37
Wang *et al.* (2016) [36]	PBE	-	VASP	-60

Supplementary Table 4. Overview of published lp-np energy differences per unit cell of MIL-53(Al).

Supplementary Note 2. Available experimental structural data for MIL-53(Al)

There is very limited high-precision low-temperature experimental data available on the shape of the potential energy surface of MIL-53(Al). Moreover, most structural data on flexible MOFs such as MIL-53(Al) is fitted to powder X-ray diffraction experiments (in contrast to single-crystal X-ray diffraction) under certain approximations, which can also influence, for instance, the reported lattice parameters. Therefore, one of the major reasons to start from a high-level technique such as RPA - which has been extensively benchmarked to high-precision experiments for various other systems - is to generate accurate benchmark data.

Experiment	*a* (Å)	*b* (Å)	*c* (Å)	*α* (°)	*β* (°)	*γ* (°)	*V* ($Å^3$)
Liu *et al.* (2008) [37]<br>Neutron Powder Diffraction (77 K)	19.121	6.607	6.871	90.0	90.0	95.5	864
Liu *et al.* (2008) [37]<br>Neutron Powder Diffraction (295 K)	19.098	6.609	7.055	90.0	90.0	95.1	887
Yot *et al.* (2014) [38]<br>X-ray Powder Diffraction (298 K)	19.056	6.559	7.160	90.0	90.0	94.7	897
Nevjestič *et al.* (2016) [39]<br>Synchrotron X-ray Powder Diffraction (298 K) (V-doped)	19.015	6.575	7.620	90.0	90.0	95.3	949

Supplementary Table 5. Overview of experimental lattice parameters for the np phase of MIL-53(Al) available in literature.

Experiment	*a* (Å)	*b* (Å)	*c* (Å)	*α* (°)	*β* (°)	*γ* (°)	*V* (Å^3)
Liu *et al.* (2008) [37]<br>Neutron Powder Diffraction (77 K)	16.91	6.624	12.67	90.0	90.0	90.0	1419
Liu *et al.* (2008) [37]<br>Neutron Powder Diffraction (295 K)	16.84	6.635	12.80	90.0	90.0	90.0	1430
Liu *et al.* (2008) [37]<br>Neutron Powder Diffraction (295 K)	16.761	6.638	12.839	90.0	90.0	90.0	1429
Yot *et al.* (2014) [38]<br>X-ray Powder Diffraction (298 K)	16.732	6.630	12.836	90.0	90.0	90.0	1424
Nanthamathee *et al.* (2015) [33]<br>Synchrotron X-ray Powder Diffraction (150 K)	16.797	6.630	12.747	90.0	90.0	90.0	1420
Nanthamathee *et al.* (2015) [33]<br>Synchrotron X-ray Powder Diffraction (300 K)	16.758	6.629	12.794	90.0	90.0	90.0	1421
Nevjestič *et al.* (2017) [40]<br>Synchrotron X-ray Powder Diffraction (455 K) (V-doped)	16.661	6.634	12.961	90.0	90.0	90.0	1432
Loiseau *et al.* (2004) [41]<br>Laboratory Powder X-ray Diffraction (550 K)	16.675	6.609	12.813	90.0	90.0	90.0	1412

Supplementary Table 6. Overview of experimental lattice parameters for the lp phase of MIL-53(Al) available in literature.

Supplementary Note 3. Sensitivity of DFT relative stability with respect to structural data

An in-depth investigation was performed of the sensitivity of the reported lp-np energy differences of MIL-53(Al) on the specific choice of the geometry, cell shape and equilibrium volume.

The equilibrium volume for all methodologies was systematically determined by fitting a Rose-Vinet equation of state to a local energy profile as a function of the volume.[42, 43] An example of such a fit is given for the np phase in Supplementary Figure 1.

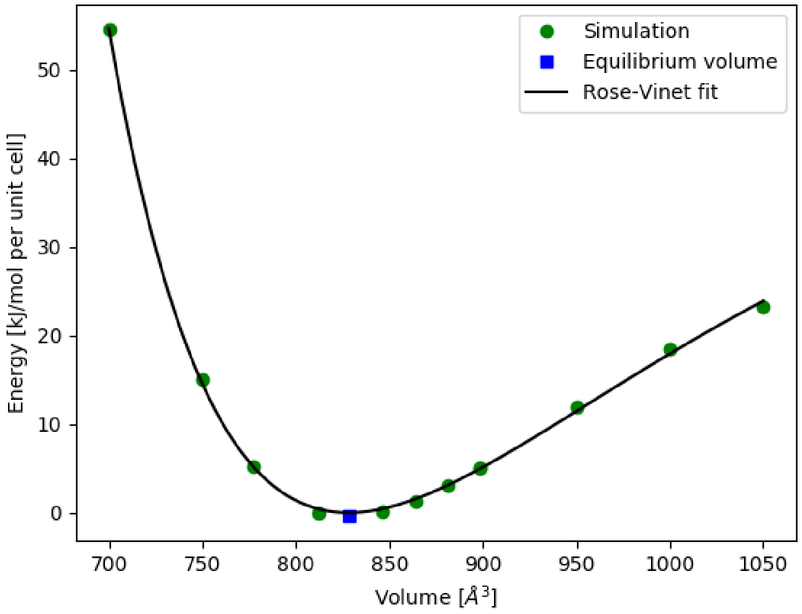

Supplementary Figure 1. An example of a local energy profile as a function of the volume obtained at the PBE+MBD level for the np phase of MIL-53(Al).

The resulting volumes are given in the main manuscript and listed in Supplementary Table 7 together with the experimentally determined values by Liu et al. [37] at 77 K. As we constructed an energy profile as a function of volume with RPA+SE, we can also extract the equilibrium volume predicted by this technique. The resulting values are also listed in Supplementary Table 7.

	V_{np} (Å^3)	V_{lp} (Å^3)	ΔE^{el}_{lp-np} (kJ·mol^{-1}) (relaxed at the corresponding level of theory)	$\Delta E^{el,*}_{lp-np}$ (kJ·mol^{-1}) (relaxed at PBE+D3(BJ) level of theory at fixed volumes of 864 Å^3 and 1427 Å^3)	$\Delta E^{el,*}_{lp-np}$ (kJ·mol^{-1}) (relaxed at the corresponding level of theory but at fixed volumes of 864 Å^3 and 1427 Å^3)
Experiment at 77 K [37]	864	1419*	-	-	-
RPA+SE	860	1455	7.4	7.7 ± 3.5	7.7 ± 3.5**
PBE+D2	808	1457	15.7	12.8	12.7
PBE+D3(BJ)	843	1426	26.6	26.1	26.1
PBE+D3(BJ)^{ATM}	874	1448	5.8	6.2	6.2
HSE06+D3(BJ)	825	lp unstable	lp unstable	42.3	42.7
HSE06+D3(BJ)^{ATM}	850	1389	22.3	22.4	22.7
B3LYP+D3(BJ)	789	lp unstable	lp unstable	58.9	58.2
B3LYP+D3(BJ)^{ATM}	806	1405	43.8	39.0	38.2
PBE+TS	793	lp unstable	lp unstable	74.2	74.3
PBE+MBD	828	1425	25.8	24.5	24.3
PBE+MBD/FI	851	1439	16.4	16.4	16.3
SCAN+rVV10	803	1384	31.2	28.3	28.0
vdW-DF2	869	1456	34.8	35.5	34.9
M06-L	829	lp unstable	lp unstable	36.6	38.4

Supplementary Table 7. The equilibrium volumes for the np and lp phase of MIL-53(Al), and their relative stability for several levels of theory. ΔE^{el}_{lp-np} is the energy difference at the equilibrium volumes and geometries of both phases relaxed with the corresponding method (or PBE+D3(BJ) for RPA+SE). $\Delta E^{el,*}_{lp-np}$ is the energy difference at fixed volumes (864 Å^3 (np) and 1427 Å^3 (lp)) with/without relaxation at the corresponding level of theory.
*The sample was going towards the np phase on a longer time scale than the measurement. [37]
**The RPA+SE structures were obtained by optimizing at the PBE+D3(BJ) level.

As the theoretical volumes were obtained at 0 K, it is advised to compare with the low-temperature results of Liu *et al*. For the np phase, a measurement at 77 K yielded a volume of 864 $Å^3$. For the porous lp phase, it is difficult to compare with low-temperature data as experimentally this phase is not stable at these temperatures. Liu *et al.* measured a lp phase, which was metastable (the sample was going towards the np phase on a longer time scale than the measurement) resulting in a volume of 1419 $Å^3$. Note also that – at higher temperatures – the experimentally measured values vary for the lp phase from 1412 $Å^3$ to 1432 $Å^3$, and for the np phase from 887 $Å^3$ to 949 $Å^3$ (Supplementary Note 2).

Our RPA+SE volume for the dense phase is in very good agreement (860 $Å^3$ vs. 864 $Å^3$) with the measurement of Liu *et al.* In contrast, most applied DFT methods underestimate the volume, but the agreement improves for schemes including long-range many-body dispersion interactions. Comparison with experimental values for the lp phase should be performed with caution, given previous remarks. We notice that our RPA+SE reference predicts a higher equilibrium volume for the lp phase (1455 $Å^3$ vs 1419 $Å^3$), while the DFT methods (not all methods predict a metastable lp phase) give values between 1384 $Å^3$ and 1457 $Å^3$ and the agreement again improves when including many-body dispersion.

In general there is some spread in the equilibrium volumes obtained with the various DFT methods and dispersion schemes. It is now important to probe the sensitivity of the energy difference between the lp and np pore phase in terms of these observed differences in equilibrium volumes and by extension to the structural data. To this end, three data sets have been generated, which allow to assess the influence of the cell structure on the relative stability of the lp and the np phase:

(a) The electronic energy difference between the lp and np phase relaxed with the corresponding DFT method. These values were already included in the main manuscript (Table 2).

(b) The energy difference between the lp and np phase relaxed with the PBE+D3(BJ) method at fixed volumes (864 $Å^3$ and 1427 $Å^3$). These values were already included in the main manuscript (Table 2).

(c) The energy difference between the lp and np phase relaxed with the corresponding DFT method but at fixed volume (864 $Å^3$ and 1427 $Å^3$). Here, the cell shape and positions were relaxed.

The results are taken up in Supplementary Table 7 (columns 4, 5 and 6). The results in (a) are obtained with different equilibrium volumes, i.e. the volumes optimized at the specific level of theory (given in columns 2 and 3), whereas the results in (b) and (c) use fixed volumes which are representative for the np and lp phase. As will follow from the results discussed below, the precise choice of the equilibrium volume does not influence our results strongly and any reasonable value for the lp phase would give to a large extent the same results due to the flat potential energy surface around the phase equilibria of these flexible materials.

A comparison of the results between cases (b) and (c) indicates that the influence of the specific unit cell shape and positions on the energy difference is limited at fixed volume (the deviation is smaller than 2 $kJ \cdot mol^{-1}$ for every method). Our single-point fixed volume approach thus enables a good estimate of the order of magnitude of the relative stability, and is ideal to quickly test the performance

of a functional and dispersion scheme in comparison to our high-level result. The unit cells at the fixed volumes are given in Supplementary Tables 8 and 9 (below).

The results of case (a) are only slightly more sensitive to the specific choice of the functional, which can be traced back to the difference in equilibrium volumes compared to the chosen fixed volumes of 864 $Å^3$ and 1427 $Å^3$. However, we remark that even in this case, the difference between (a) and (b) is always lower than 6 $kJ{\cdot}mol^{-1}$ and for most methods even lower than 2 $kJ{\cdot}mol^{-1}$. Unfortunately, for some functionals, such as HSE06+D3(BJ), B3LYP+D3(BJ), PBE+TS and M06-L, no stable large pore phase can be obtained, which makes a comparison of the relative energy differences impossible using only data set (a).

The results above indicate that there is only a limited influence of the structure on the relative energy difference between the lp and np phase.

	a (Å)	*b* (Å)	*c* (Å)	*α* (°)	*β* (°)	*γ* (°)
PBE+D2	19.253	6.667	6.760	90.0	90.0	95.4
PBE+D3(BJ)	19.246	6.661	6.765	90.0	90.0	95.0
HSE06+D3(BJ)	19.068	6.608	6.897	90.0	90.0	94.7
B3LYP+D3(BJ)	19.147	6.629	6.831	90.0	90.0	94.8
PBE+TS	19.250	6.672	6.752	90.0	90.0	95.1
PBE+MBD	19.205	6.641	6.799	90.1	90.0	94.9
PBE+MBD/FI	19.232	6.651	6.781	90.0	90.0	95.1
SCAN+rVV10	19.025	6.600	6.914	90.0	90.0	95.7
vdW-DF2	19.411	6.719	6.650	90.0	90.0	95.0
M06-L	18.935	6.593	6.967	90.0	90.0	96.6

Supplementary Table 8. A comparison of unit cell parameters between different methods in the optimized structure of the np phase of MIL-53(Al) (at 864 $Å^3$).

	a (Å)	*b* (Å)	*c* (Å)	*α* (°)	*β* (°)	*γ* (°)
PBE+D2	17.128	6.672	12.493	90.0	90.0	91.0
PBE+D3(BJ)	17.079	6.663	12.545	90.0	90.0	90.8
HSE06+D3(BJ)	16.550	6.625	13.019	90.0	90.0	90.4
B3LYP+D3(BJ)	16.710	6.631	12.883	90.0	90.0	90.6
PBE+TS	17.108	6.665	12.521	90.0	90.0	90.8
PBE+MBD	16.963	6.649	12.657	90.0	90.0	90.7
PBE+MBD/FI	17.054	6.657	12.574	90.0	90.0	90.8
SCAN+rVV10	16.431	6.622	13.120	90.0	90.0	90.7
vdW-DF2	17.548	6.735	12.080	90.0	90.0	91.0
M06-L	16.373	6.639	13.134	90.0	90.0	91.0

Supplementary Table 9. A comparison of unit cell parameters between different methods in the optimized structure of the lp phase of MIL-53(Al) (at 1427 Å^3).

As many-body dispersion interactions seem to have an influence on the equilibrium volume, we also investigated the influence of these effects on the internal coordinates of the crystal structure. Different dispersion schemes with/without many-body effects on top of PBE (TS, MBD, MBD/FI, D3(BJ)), and two other functionals (HSE06+D3(BJ) and vdW-DF2) are compared in Supplementary Tables 10 - 15. In these tables, some important internal coordinates (bond lengths, bend angles, dihedral angles) and unit cell parameters are reported for various methods (obtained at fixed volumes of the np and lp phase). The definition of the atom types is displayed in Supplementary Figure 2.

It can be seen that the difference between PBE and the other two functionals is generally larger than the effects of the dispersion schemes on the geometry. This is in contrast with the big dependence of the relative stability on the dispersion method. Especially interesting is the comparison between the PBE+TS (pairwise) and PBE+MBD (many-body effects) level for which the influence of many-body effects on the geometry can directly be probed. The results indicate that many-body effects do not have much influence on the geometry of the structure, which can also be seen in the reported unit cell parameters (see also Supplementary Tables 8 and 9). This analysis also suggests that the ATM corrections will not have a large influence on the unit cell shape and/or internal coordinates, as claimed previously in the literature.[24]

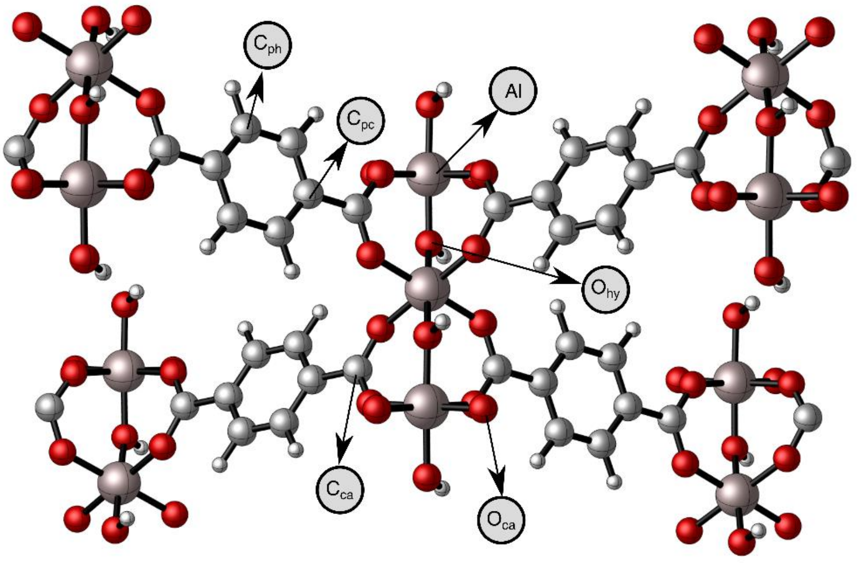

Supplementary Figure 2. Definition of the atom types in MIL-53(Al) used to indicate the internal coordinates.

Bond length (Å)	PBE+TS	PBE+MBD	PBE+MBD/FI	PBE+D3(BJ)	HSE06+D3(BJ)	vdW-DF2
Al-O_{ca}	1.92±0.01	1.91±0.01	1.92±0.01	1.92±0.01	1.90±0.01	1.94±0.01
Al-O_{hy}	1.86±0.00	1.85±0.00	1.86±0.00	1.86±0.00	1.84±0.00	1.88±0.00
O_{ca}-C_{ca}	1.27±0.00	1.27±0.00	1.27±0.00	1.27±0.00	1.26±0.00	1.28±0.00
C_{ca}-C_{pc}	1.49±0.00	1.49±0.00	1.49±0.00	1.49±0.00	1.49±0.00	1.50±0.00
C_{pc}-C_{ph}	1.40±0.00	1.40±0.00	1.40±0.00	1.40±0.00	1.39±0.00	1.41±0.00
C_{ph}-C_{ph}	1.39±0.00	1.39±0.00	1.39±0.00	1.39±0.00	1.38±0.00	1.39±0.00

Supplementary Table 10. A comparison of different bond lengths between different methods in the optimized structure of the np phase of MIL-53(Al) (at 864 Å^3). The definition of atom types is given in Supplementary Figure 2.

Bond length (Å)	PBE+TS	PBE+MBD	PBE+MBD/FI	PBE+D3(BJ)	HSE06+D3(BJ)	vdW-DF2
Al-O_{ca}	1.92±0.01	1.91±0.01	1.92±0.01	1.92±0.01	1.90±0.01	1.94±0.01
Al-O_{hy}	1.86±0.00	1.85±0.00	1.86±0.00	1.86±0.00	1.84±0.00	1.87±0.00
O_{ca}-C_{ca}	1.27±0.00	1.27±0.00	1.27±0.00	1.27±0.00	1.26±0.00	1.28±0.00
C_{ca}-C_{pc}	1.49±0.00	1.49±0.00	1.49±0.00	1.49±0.00	1.49±0.00	1.50±0.00
C_{pc}-C_{ph}	1.40±0.00	1.40±0.00	1.40±0.00	1.40±0.00	1.39±0.00	1.41±0.00
C_{ph}-C_{ph}	1.39±0.00	1.39±0.00	1.39±0.00	1.39±0.00	1.38±0.00	1.39±0.00

Supplementary Table 11. A comparison of different bond lengths between different methods in the optimized structure of the lp phase of MIL-53(Al) (at 1427 Å^3). The definition of atom types is given in Supplementary Figure 2.

Bend angle (°)	PBE+TS	PBE+MBD	PBE+MBD/FI	PBE+D3(BJ)	HSE06+D3(BJ)	vdW-DF2
O_{ca}-Al-O_{ca}	120.0±42.5	120.0±42.5	120.0±42.5	120.0±42.5	120.0±42.5	120.0±42.5
O_{ca}-C_{ca}-O_{ca}	124.6±0.0	124.5±0.0	124.5±0.0	124.6±0.0	124.5±0.0	124.1±0.0
Al-O_{hy}-Al	127.0±0.0	127.1±0.0	126.9±0.0	127.0±0.0	127.3±0.0	127.0±0.0
O_{hy}-Al-O_{hy}	179.9±0.0	179.9±0.0	180.0±0.0	180.0±0.0	180.0±0.0	180.0±0.0
C_{ph}-C_{pc}-C_{ph}	119.8±0.0	119.8±0.0	119.7±0.0	119.8±0.0	120.0±0.0	119.5±0.0

Supplementary Table 12. A comparison of different bend angles between different methods in the optimized structure of the np phase of MIL-53(Al) (at 864 Å^3). The definition of atom types is given in Supplementary Figure 2.

Bend angle (°)	PBE+TS	PBE+MBD	PBE+MBD/FI	PBE+D3(BJ)	HSE06+D3(BJ)	vdW-DF2
O_{ca}-Al-O_{ca}	119.6±41.9	119.7±42.0	119.7±42.0	119.7±42.0	119.8±42.1	119.9±42.3
O_{ca}-C_{ca}-O_{ca}	125.5±0.1	125.5±0.1	125.5±0.1	125.5±0.1	125.5±0.1	125.0±0.0
Al-O_{hy}-Al	127.2±0.0	127.4±0.0	127.4±0.0	127.4±0.0	128.1±0.0	128.0±0.0
O_{hy}-Al-O_{hy}	177.8±0.0	178.4±0.0	178.3±0.0	178.3±0.0	178.7±0.0	179.5±0.0
C_{ph}-C_{pc}-C_{ph}	119.8±0.1	119.8±0.1	119.7±0.1	119.8±0.1	119.9±0.1	129.5.±0.0

Supplementary Table 13. A comparison of different bend angles between different methods in the optimized structure of the lp phase of MIL-53(Al) (at 1427 Å^3). The definition of atom types is given in Supplementary Figure 2.

Dihedral angle (°)	PBE+TS	PBE+MBD	PBE+MBD/FI	PBE+D3(BJ)	HSE06+D3(BJ)	vdW-DF2
C_{ph}-C_{pc}-C_{ca}-O_{ca}	88.8±86.5	89.0±86.4	89.0±86.4	89.1±86.3	89.6±86.1	88.2±86.7
Al-O_{ca}-C_{ca}-C_{pc}	140.2±14.4	140.5±14.3	140.3±14.7	140.2±14.5	140.7±13.9	139.2±14.3
Al-O_{ca}-C_{ca}-O_{ca}	36.4±14.4	36.0±14.4	36.5±14.8	36.5±14.5	36.3±13.9	36.9±14.3

Supplementary Table 14. A comparison of different dihedral angles between different methods in the optimized structure of the np phase of MIL-53(Al) (at 864 Å^3). The definition of atom types is given in Supplementary Figure 2.

Dihedral angle (°)	PBE+TS	PBE+MBD	PBE+MBD/FI	PBE+D3(BJ)	HSE06+D3(BJ)	vdW-DF2
C_{ph}-C_{pc}-C_{ca}-O_{ca}	90.0±86.1	90.0±87.5	90.0±87.6	90.0±86.9	90.0±86.6	90.0±88.5
Al-O_{ca}-C_{ca}-C_{pc}	169.1±8.3	169.8±7.9	169.2±8.8	169.0±9.1	171.7±7.6	165.8±8.0
Al-O_{ca}-C_{ca}-O_{ca}	10.9±8.3	10.2±7.9	10.8±8.8	11.0±9.1	8.5±7.8	14.0±8.0

Supplementary Table 15. A comparison of different dihedral angles between different methods in the optimized structure of the lp phase of MIL-53(Al) (at 1427 Å^3). The definition of atom types is given in Supplementary Figure 2.

We also performed single-point calculations on different structures of the dense np phase - which is dispersion dominated - using PBE+D3(BJ) and PBE+D3(BJ)^{ATM}. The different structures are the ones reported in Supplementary Table 8. The energy differences displayed in Supplementary Figure 3 are thus a measure of the ATM corrections for various optimized geometries. The results show that the relative energies or the correction obtained by using the three-body ATM terms is rather insensitive to the specific structure (differences smaller than 1 kJ·mol^{-1} for the absolute np phase energy). This gives further evidence that small corrections to the unit cell will not influence our findings.

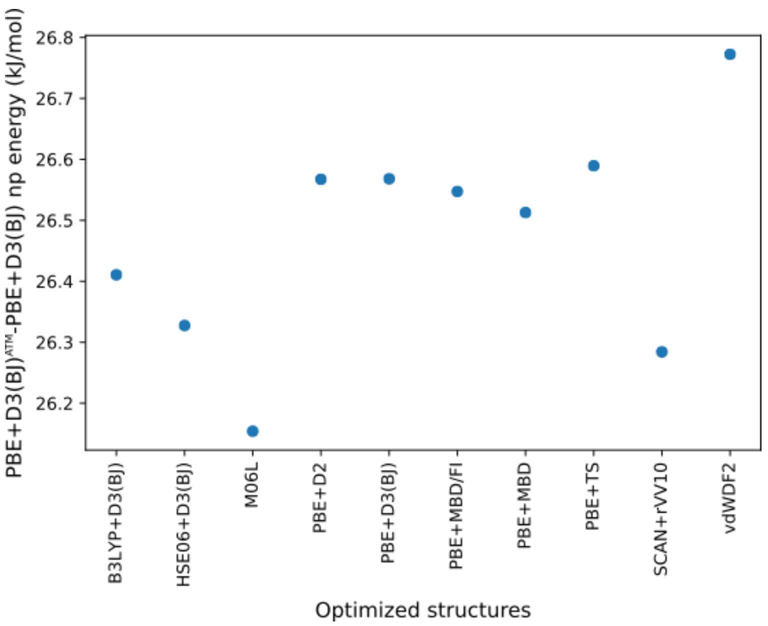

Supplementary Figure 3. Absolute energy difference between PBE+D3(BJ)^{ATM} and PBE+D3(BJ) (or thus the ATM corrections) for different geometries of MIL-53(Al) (at a fixed volume of 864 Å^3) obtained with different levels of theory.

Summarizing, this analysis shows that many-body dispersion effects have a smaller impact on the geometries than the choice of the particular exchange-correlation (XC) functional. Furthermore, the observed small changes in the structure due to many-body dispersion effects induce only very small energy differences.

Supplementary Note 4. Sensitivity of DFT relative stability on the coupling between the exchange-correlation functional and the dispersion scheme

To systematically investigate the impact of both the XC functional and the dispersion method, we included other functionals with various dispersion schemes encompassing both pair-wise and many-body dispersion schemes. Supplementary Table 16 lists the electronic energy difference between the lp and np phase of MIL-53(Al) with different methods at fixed geometries obtained with the PBE-D3(BJ) level of theory (Supplementary Tables 8 and 9). The values reported in the main article are indicated in bold.

	XC	XC+D3(BJ)	XC+D3(BJ)^{ATM}	XC+TS	XC+MBD
PBE	**-118.5**	**26.1**	**6.2**	**74.2**	**24.5**
TPSS	-141.6	43.3	23.5	/	/
SCAN	**-43.6**	33.7 [44]	13.8	/	48.5 (β=1.09)<br>36.0 (β=1.16)<br>25.3 (β=1.23) [45]
HSE06	-111.7	**42.3** [24]	**22.4**	75.3 [46]	28.0 [47]
PBE0	-110.8	37.7	17.9	76.2 [48]	28.9
B3LYP	-145.1	**58.9**	**39.0**	/	/

Supplementary Table 16. The electronic energy difference between the lp and np phase of MIL-53(Al) with different methods. It is the energy difference at fixed structures (np: 864 Å^3, lp: 1427 Å^3) relaxed with PBE+D3(BJ). The bold numbers are also indicated in the main manuscript (Table 2). The references to the coupling parameters between the XC functional and dispersion scheme that cannot be found in the original D3(BJ), TS and MBD papers are indicated in the table.

Our results for the TS and MBD scheme (columns 5 and 6) show that irrespective of the XC functional or dispersion method, a similar lp-np energy difference is obtained. The only exception is SCAN, which suffers from a sensitive coupling with dispersion schemes.[45] An entirely different situation is obtained when considering D3(BJ) and D3(BJ)^{ATM} dispersion schemes. In that case, changing the XC functional drastically changes the computed phase stability, raising the energy differences by 20 to 30 kJ·mol^{-1}. The different behavior of D3- and TS-based schemes is due to the coupling between the functional and the dispersion method.

A striking observation is that going from PBE (a local GGA) to meta-GGA (TPSS, SCAN) or hybrid functionals (HSE06, PBE0, B3LYP) worsens the agreement with RPA+SE. In Supplementary Table 17, the results are summarized for PBE and PBE0 and show that the difference Δ between the lp-np energy difference according to PBE and PBE0 coupled with a D3 scheme comes to a large extent from the XC energy difference between PBE and PBE0 and not from the dispersion scheme. The disagreement between PBE0+D3(BJ) and RPA+SE may therefore be due to either an unsuitability of the hybrid functional or, more likely, an insufficiently sensitive fitting of the D3 dispersion parameters to the parent functional. Indeed, the difference between PBE and PBE0 for the TS and MBD schemes is more

subtle and is partially due to the XC energy difference and partially due to the dispersion energy difference, resulting in its previously ascribed robustness. While D3(BJ) predicts a difference in dispersion energy between the lp and np phase that is slightly larger for PBE0 than PBE, TS and MBD display the opposite behavior.

	XC	XC+D3(BJ)	XC+D3(BJ)^{ATM}	XC+TS	XC+MBD
PBE	-118.5	26.1	6.2	74.2	24.5
PBE0	-110.8	37.7	17.9	76.2	28.9
Δ	-7.7	-11.6	-11.7	-2.0	-4.4

Supplementary Table 17. The electronic energy difference between the lp and np phase of MIL-53(Al) with different methods. It is the energy difference at fixed structures (np: 864 Å^3, lp: 1427 Å^3) relaxed with PBE+D3(BJ). The fourth row (Δ) corresponds to the difference between the second (PBE) and third row (PBE0) and represents the sensitivity of the lp-np energy difference to the XC functional.

Although the TS or MBD schemes provide a much more robust coupling between the XC functional and the dispersion method, their use does not improve the agreement with the RPA+SE value of 7.7 $\pm$ 3.5 kJ·mol^{-1} for this specific system. Possibly, these results indicate that in case of a hybrid porous material there might be a problem when transferring a model fitted to small and medium-size dimers to large unit cells containing many different bond types simultaneously.

Supplementary Note 5. Selection of a proper geometry for the RPA+SE calculations

Because it is currently computationally not feasible to perform structural relaxations of MIL-53(Al) at the RPA level, RPA calculations were performed using DFT-optimized crystal structures. PBE+D3(BJ) was chosen as a suitable method to produce these structures, as it provides a good estimate of both geometric and energetic features. Indeed, the PBE+D3(BJ)^{ATM} lp-np energy difference and equilibrium volumes of both phases are in good agreement with RPA+SE results (Supplementary Table 7). In addition, Supplementary Tables 10-15 show that the internal coordinates describing the atomic geometry are only negligibly dependent on the level of theory used in the optimization. Finally, Supplementary Table 18 proves that different input geometries only marginally affect the RPA+SE lp-np energy difference.

	E_{np} (kJ·mol^{-1})	E_{lp} (kJ·mol^{-1})	$\Delta E^{el,*}_{lp-np}$ (kJ·mol^{-1})
RPA+SE<br>at PBE-D3(BJ) optimized geometries for fixed volumes of 864 Å^3 (np) and 1427 Å^3 (lp)	0	7.7	7.7 ± 3.5
RPA+SE<br>at HSE06-D3(BJ) optimized geometries for fixed volumes of 864 Å^3 (np) and 1427 Å^3 (lp)	60.0	66.2	6.2
RPA+SE<br>at vdW-DF2 optimized geometries for fixed volumes of 864 Å^3 (np) and 1427 Å^3 (lp)	-3.5	6.5	10.0

Supplementary Table 18. The electronic energy difference between the lp and np phase of MIL-53(Al) at the RPA+SE level of theory based on geometries determined with different electronic-structure methods. Absolute energies are expressed relative to the result for RPA+SE on top of the np PBE+D3(BJ) geometry.

Supplementary Table 18 compares structures relaxed with three different methods (PBE+D3(BJ), HSE06+D3(BJ) and vdW-DF2). These methods were selected to incorporate two different classes of XC functionals (GGA and hybrid functionals) and two types of dispersion approaches (a posteriori corrections and contributions included in the functional itself). In addition, these three functionals yield some of the strongest changes in np cell shape (Supplementary Tables 8 and 9). While absolute RPA+SE energies vary by up to 60 kJ·mol^{-1}, we found the relative stability to remain within the RPA+SE error bar of 3.5 kJ·mol^{-1} (see Methods section of the main manuscript). This is because small differences in the *b* parameter, which is related to the stiff metal-oxide bonds, yield relatively large differences in absolute RPA+SE energy. However, each of the three considered electronic-structure methods affects these bonds in both the lp and np phase to a similar extent, resulting in an overall negligible difference in lp-np phase stability. Therefore, we can safely assume that our results obtained using PBE+D3(BJ) geometries are only slightly sensitive to the precise level of theory used for the geometries.

Supplementary Note 6. Finite-temperature corrections with DFT

The spread on the finite-temperature corrections in Figure 3 (main article) results from different predicted phonon densities of states, showing that these predictions are also sensitive to the chosen method. As thermal corrections at the RPA+SE level for large systems like MOFs are not possible using the available current computational resources, we cannot benchmark our DFT predictions to a higher-level method, which is why we included all predictions to indicate the sensitivity. However, for this specific system, some of us recently investigated the experimental infrared and Raman spectrum of the np and lp phase of MIL-53(Al) and showed a good agreement with our PBE+D3(BJ) predicted spectrum.[49]

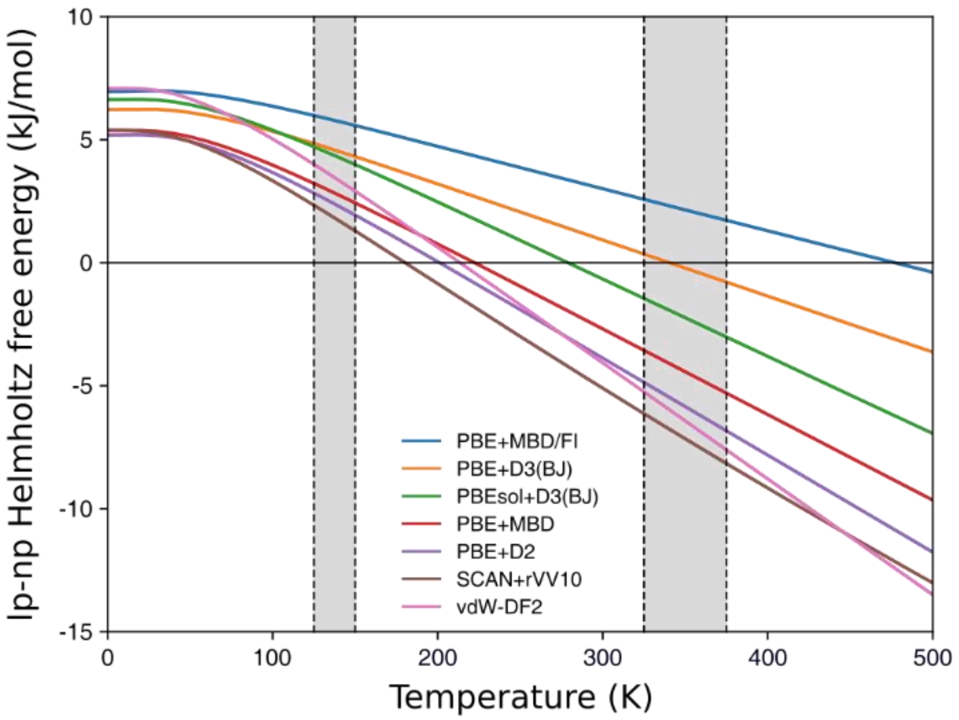

Supplementary Figure 4. Finite-temperature corrections for the lp-np energy difference of MIL-53(Al) with DFT on top of the RPA+SE electronic energy difference.

Supplementary Note 7. RPA(+SE) energy profile as a function of the volume

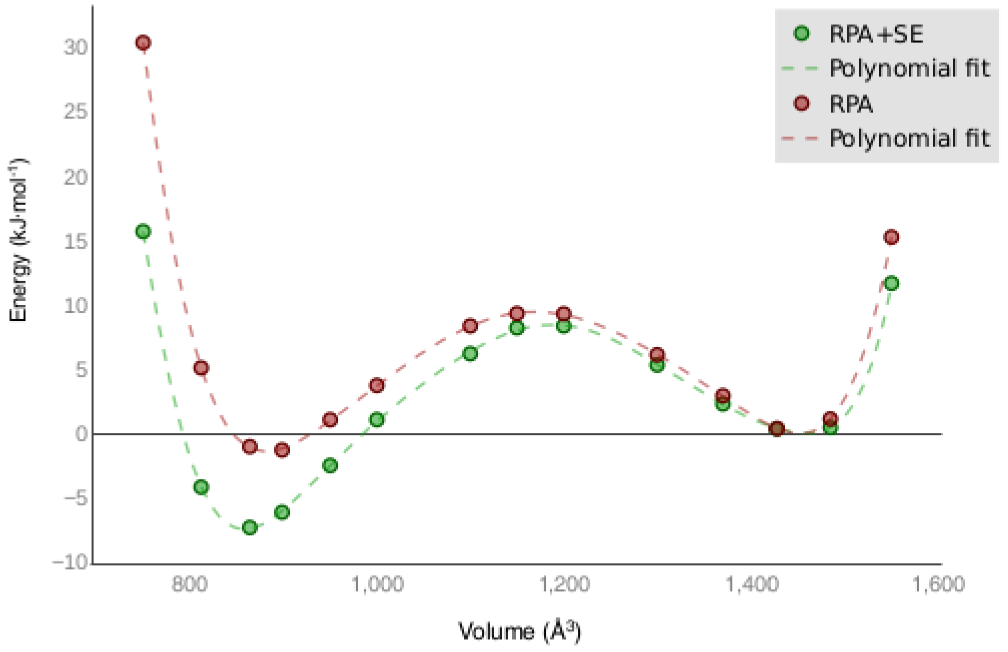

Supplementary Figure 5. MIL-53(Al) energy profile as a function of the volume with RPA and RPA+SE illustrating the importance of the inclusion of the SE contribution.

Supplementary Note 8. Structures

An overview is provided of the materials considered in this work and their names used in literature in Supplementary Table 19. The optimized geometries for the series of isoreticular MIL-53(Al) materials used to calculate the Helmholtz free energy difference between the np and lp phase as a function of temperature are given in Supplementary Table 20 and are compared to the experimental structures.

Material	*Inorganic part*	*Organic Linker*	*Chemical Name*	*Synonyms*
MIL-53(Al)	Al(OH) 1D chain	1,4-benzene dicarboxylate ($H_4C_8O_4$, BDC)	Al(OH)(BDC)	MIL-53(Al)-BDC, Aluminum terephthalate, Basolite A100
MIL-53(Ga)	Ga(OH) 1D chain	1,4-benzene dicarboxylate ($H_4C_8O_4$, BDC)	Ga(OH)BDC	MIL-53(Ga)-BDC, Gallium terephthalate, IM-19
MIL-53(Al)-FA	Al(OH) 1D chain	fumaric acid ($H_2C_4O_4$, FA)	Al(OH)(FA)	A520, Aluminum fumarate
DUT-4	Al(OH) 1D chain	2,6-napthalene dicarboxylate ($H_6C_{12}O_4$, NDC)	Al(OH)(NDC)	MIL-69, MIL-53(Al)-NDC
DUT-5	Al(OH) 1D chain	4,4'-biphenyl dicarboxylate ($H_8C_{14}O_4$, BPDC)	Al(OH)(BPDC)	MIL-53(Al)-BPDC

Supplementary Table 19. Overview of considered materials and their names used in literature.

Material	*Phase*	*a (Å)*	*b (Å)*	*c (Å)*	*α (°)*	*β (°)*	*γ (°)*	*V (Å³)*
MIL-53(Al)	np	19.252	6.655	6.606	90.0	90.0	95.3	843
	np (exp. at 77 K) [37]	19.121	6.607	6.871	90.0	90.0	95.5	864
	lp	17.089	6.662	12.528	90.0	90.0	90.8	1,426
	lp (exp. at 77 K) [37]	16.91	6.624	12.67	90.0	90.0	90.0	1,419
MIL-53(Ga)	np	19.522	6.771	6.387	97.4	92.3	96.3	831
	np (exp. at 413 K) [50]	19.302	6.716	7.158	90.0	90.0	95.1	924
	lp	17.018	6.739	13.166	90.0	90.0	90.4	1,510
	lp (exp. at 623 K) [50]	16.734	6.741	13.282	90.0	90.0	90.0	1,498
MIL-53(Al)-FA	np	15.054	6.720	6.285	107.7	90.0	90.0	606
	np (exp. at 298 K and 410 MPa) [51]	15.120	6.630	7.676	103.4	90.0	90.0	750
	lp	13.449	6.688	10.744	101.61	90.0	90.0	947
	lp (exp. at 298 K) [51]	12.154	7.022	11.846	99.6	90.0	90.0	998
DUT-4	np	23.697	6.639	6.974	90.0	90.0	91.0	1,097
	np (exp. at 298 K with H_2O) [41]	24.625	6.560	7.528	90.0	90.0	91.4	1,160
	lp*	19.633	6.683	16.550	90.0	90.0	96.2	2,159
	lp (exp. at 293 K) [52]	18.825	6.787	16.901	90.0	90.0	90.0	2,159
DUT-5	np	27.852	6.643	6.628	98.2	92.5	96.2	1,205
	lp	24.509	6.653	17.044	90.0	90.0	90.2	2,779
	lp (exp. at 293 K) [52]	22.698	6.607	19.240	90.0	90.0	90.0	2,885

Supplementary Table 20. Crystallographic data of a series of isoreticular MIL-53(Al) materials. The equilibrium volume was obtained by fitting the Rose-Vinet equation of state [42] to a local PBE+D3(BJ) energy profile as a function of volume.
*The lp phase of DUT-4 is not a minimum on the potential energy surface as a function of volume at the PBE+D3(BJ) level and was optimized at the experimental volume.

Supplementary References

1. Perdew, J. P., Burke, K. & Ernzerhof, M. Generalized gradient approximation made simple. Phys. Rev. Lett. **77**, 3865 (1996).
2. Perdew, J. P., Burke, K. & Ernzerhof, M. Generalized gradient approximation made simple [Phys. Rev. Lett. 77, 3865 (1996)]. Phys. Rev. Lett. **78**, 1396 (1997).
3. Perdew, J. P. et al. Restoring the density-gradient expansion for exchange in solids and surfaces. Phys. Rev. Lett. **100**, 136406 (2008).
4. Perdew, J. P. et al. Erratum: Restoring the density-gradient expansion for exchange in solids and surfaces [Phys. Rev. Lett. 100, 136406 (2008)]. Phys. Rev. Lett. **102**, 039902 (2009).
5. Dion, M., Rydberg, H., Schröder, E., Langreth, D. C. & Lundqvist, B. I. Van der Waals density functional for general geometries. Phys. Rev. Lett. **92**, 246401 (2004).
6. Klimeš, J., Bowler, J. R. & Michaelides, A. Van der Waals density functionals applied to solids. Phys. Rev. B **83**, 195131 (2011).
7. Lee, K., Murray, É. D., Kong, L., Lundqvist, B. I. & Langreth, D. C. Higher-accuracy van der Waals density functional. Phys. Rev. B **82**, 081101 (2010).
8. Román-Peréz, G. & Soler, J. M. Efficient implementation of a van der Waals density functional: application to double-wall carbon nanotubes. Phys. Rev. Lett. **103**, 096102 (2009).
9. Sun, J. et al. Accurate first-principles structures and energies of diversely bonded systems from an efficient density functional. Nat. Chem. **8**, 831-836 (2016).
10. Sun, J., Ruzsinszky, A. & Perdew, J. P. Strongly constrained and appropriately normed semilocal density functional. Phys. Rev. Lett. **115**, 036402 (2015).
11. Peng, H., Yang, Z.-H., Perdew, J. P. & Sun, J. Versatile van der Waals density functional based on a meta-generalized gradient approximation. Phys. Rev. X **6**, 041005 (2016).
12. Sabatini, R., Gorni, T. & de Gironcoli, S. Nonlocal van der Waals density functional made simple and efficient. Phys. Rev. B **87**, 041108(R) (2013).
13. Vydrov, O. A. & Van Voorhis, T. Nonlocal van der Waals density functional: the simpler the better. J. Chem. Phys. **133**, 244103 (2010).
14. Zhao, Y. & Truhlar, D. G. A new local density functional for main-group thermochemistry, transition metal bonding, thermochemical kinetics, and noncovalent interactions. J. Chem. Phys. **125**, 194101 (2006).
15. Becke, A.D. Density-functional exchange-energy approximation with correct asymptotic behavior. Phys. Rev. A **38**, 3098-3100 (1988).
16. Becke, A. D. Density-gunctional thermochemistry. III. The role of exact exchange. J. Chem. Phys. **98**, 5648-5652 (1993).
17. Lee, C., Yang, W. & Parr, R. G. Development of the Colle-Salvetti correlation-energy formula into a functional of the electron density. Phys. Rev. B **37**, 785-789 (1988).
18. Vosko, S. H., Wilk, L. & Nusair, M. Accurate spin-dependent electron liquid correlation energies for local spin density calculations: A critical analysis. Can. J. Phys. **58**, 1200-1211 (1980).
19. Heyd, J., Scuseria, G. E. & Ernzerhof, M. Hybrid functionals based on a screened Coulomb potential. J. Chem. Phys. **118**, 8207 (2003).
20. Heyd, J., Scuseria, G. E. & Ernzerhof, M. Erratum: "Hybrid functionals based on a screened Coulomb potential" [J. Chem. Phys. 118, 8207 (2003)]. J. Chem. Phys. **124**, 219906 (2006).
21. Krukau, A. V., Vydrov, O. A., Izmaylov, A. F. & Scuseria, G. E. Influence of the exchange screening parameter on the performance of screened hybrid functionals. J. Chem. Phys. **125**, 224106 (2006).
22. Grimme, S., Antony, J., Ehrlich, S. & Krieg, H. A consistent and accurate ab initio parametrization of density functional dispersion correction (DFT-D) for the 94 Elements H-Pu. J. Chem. Phys. **132**, 15104 (2010).
23. Grimme, S., Ehrlich, S. & Goerigk, L. Effect of the damping function in dispersion corrected density functional theory. J. Comput. Chem. **132**, 1456-1465 (2011).

24. Moellmann, J. & Grimme, S. DFT-D3 study of some molecular crystals. J. Phys. Chem. C **118**, 7615-7621 (2014).
25. Grimme, S. Semiempirical GGA-type density functional constructed with a long-range dispersion correction. J. Comput. Chem. **27**, 1787-1799 (2006).
26. Tkatchenko, A. & Scheffler, M. Accurate molecular van der Waals interactions from ground-state electron density and free-atom reference data. Phys. Rev. Lett. **102**, 073005 (2009).
27. Ambrosetti, A., Reilly, A. M., DiStasio Jr., R. A. & Tkatchenko, A. Long-range correlation energy calculated from coupled atomic response functions. J. Chem. Phys. **140**, 18A508 (2014).
28. Bučko, T., Lebègue, S., Gould, T. & Ángyán, J. G. Many-body dispersion corrections for periodic systems: An efficient reciprocal space implementation. J. Phys. Condens. Matter. **28**, 045201 (2016).
29. DiStasio Jr., R. A., Gobre, V. V. & Tkatchenko, A. Many-body van der Waals interactions in molecules and condensed matter. J. Phys. Condens. Matter **26**, 213202 (2014).
30. Tkatchenko, A., DiStasio Jr., R. A., Car, R. & Scheffler, M. Accurate and efficient method for many-body van der Waals interactions. Phys. Rev. Lett. **108**, 236402 (2012).
31. Gould, T., Lebègue, S., Ángyán, J. G. & Bučko, T. A fractionally ionic approach to polarizability and van der Waals many-body dispersion calculations. J. Chem. Theory Comput. **12**, 5920-5930 (2016).
32. Walker, A. M. et al. Flexibility in a metal-organic framework controlled by weak dispersion forces: the bistability of MIL-53(Al). Angew. Chem. Int. Ed. **49**, 7501-7503 (2010).
33. Nanthamathee, C., Ling, S., Slater, B. & Attfield, M. P. Contradistinct thermoresponsive behavior of isostructural MIL-53 type metal-organic frameworks by modifying the framework inorganic anion. Chem. Mater **27**, 85-95 (2015).
34. Ling, S. & Slater, B. Unusually large band gap changes in breathing metal-organic framework materials. J. Phys. Chem. C **119**, 16667-16677 (2015).
35. Coudert, F.-X., Boutin, A. & Fuchs, A. H. A thermodynamic description of the adsorption-induced structural transitions in flexible MIL-53 metal-organic framework. Mol. Phys. **112**, 1257-1261 (2014).
36. Wang, M., Zhang, X. & Li, D. How guest molecules stabilize the narrow pore phase of soft porous crystals: structural and mechanical properties of MIL-53(Al)$\supset H_2O$. J. Phys. Chem. C **120**, 5059-5066 (2016).
37. Liu, Y. et al. Reversible structural transition in MIL-53 with large temperature hysteresis. J. Am. Chem. Soc. **130**, 11813-11818 (2008).
38. Yot, P. G. et al. Metal-organic frameworks as potential shock absorbers: the case of the highly flexible MIL-53(Al). Chem. Commun. **50**, 9462-9464 (2014).
39. Nevjestič, I. et al. In situ electron paramagnetic resonance and X-ray diffraction monitoring of temperature-induced breathing and related structural transformations in activated V-doped MIL-53(Al). J. Phys. Chem. C **120**, 17400-17407 (2016).
40. Nevjestič, I. et al. Sensing the framework state and guest molecules in MIL-53(Al) via the electron paramagnetic resonance spectrum of V^{IV} dopant ions. Phys. Chem. Chem. Phys. **19**, 24545-24554 (2017).
41. Loiseau, T. et al. A rationale for the large breathing of the porous aluminum terephthalate (MIL-53) upon hydration. Chem.–Eur. J. **10**, 1373-1382 (2004).
42. Vinet, P., Ferrante, J., Rose, J. H. & Smith, J. R. Compressibility of solids. J. Geophys. Res. **92**, 9319-9325 (1987).
43. Vanpoucke, D. E. P., Lejaeghere, K., Van Speybroeck, V., Waroquier, M. & Ghysels, A. Mechanical properties from periodic plane wave quantum mechanical codes: the challenge of the flexible nanoporous MIL-47(V) framework. J. Phys. Chem. C **119**, 23752-23766 (2015).
44. Brandenburg, J. G., Bates, J. E., Sun, J. & Perdew, J. P. Benchmark tests of a strongly constrained semilocal functional with a long-range dispersion correction. Phys. Rev. B **94**, 115144 (2016).

45. Hermann, J. & Tkatchenko, A. Electronic exchange and correlation in van der Waals systems: balancing semilocal and nonlocal energy contributions. J. Chem. Theory Comput. **14**, 1361-1369 (2018).
46. Zhang, G.-X., Tkatchenko, A., Paier, J., Appel, H. & Scheffler, M. Van der Waals interactions in ionic and semiconductor solids. Phys. Rev. Lett. **107**, 245501 (2011).
47. Maurer, R. J., Ruiz, V. G. & Tkatchenko, A. Many-body dispersion effects in the binding of adsorbates on metal surfaces. J. Chem. Phys. **143**, 102808 (2015).
48. Marom, N., Tkatchenko, A., Scheffler, M. & Kronik, L. Describing both dispersion interactions and electronic structure using density functional theory: the case of metal-phthalocyanine dimers. J. Chem. Theory Comput. **6**, 81-90 (2010).
49. Hoffman, A. E. J. et al. Elucidating the vibrational fingerprint of the flexible metal-organic framework MIL-53(Al) using a combined experimental/computational approach. J. Phys. Chem. C **122**, 2734-2746 (2018).
50. Chaplais, G. et al. IM-19: a new flexible microporous gallium based-MOF framework with pressure- and temperature-dependent openings. Phys. Chem. Chem. Phys. **11**, 5241-5245 (2009).
51. Yot, P. G. et al. Mechanical energy storage performance of an aluminum fumarate metal-organic framework. Chem. Sci. **7**, 446-450 (2016).
52. Senkovska, I. et al. New highly porous aluminium based metal-organic frameworks: Al(OH)(NDC) (NDC = 2,6-naphthalene dicarboxylate) and Al(OH)(BPDC) (BPDC = 4,4'-biphenyl dicarboxylate). Micropor. Mesopor. Mat. **122**, 93-98 (2009).
