# Supplementary material for: Tuning the balance between dispersion and entropy to design temperature-responsive flexible metal-organic frameworks
Source: Nat Commun. 2018 Nov 21;9:4899. doi: 10.1038/s41467-018-07298-4 (PMC6249296; doi:10.1038/s41467-018-07298-4)
Supplement: Supplementary file 2 — Description of Additional Supplementary Files [file 41467_2018_7298_MOESM2_ESM.docx]

**Description of Additional Supplementary Files**

**File Name:** Supplementary Data 1

**Description:** The large-pore phase crystallographic unit cell of MIL-53(Al) obtained at the PBE+D3(BJ) level of theory

**File Name:** Supplementary Data 2

**Description:** The narrow-pore phase crystallographic unit cell of MIL-53(Al) obtained at the PBE+D3(BJ) level of theory.
