# Supplementary material for: Tuning the balance between dispersion and entropy to design temperature-responsive flexible metal-organic frameworks
Source: Nat Commun. 2018 Nov 21;9:4899. doi: 10.1038/s41467-018-07298-4 (PMC6249296; doi:10.1038/s41467-018-07298-4)
Supplement: Supplementary file 3 — Supplementary Data 1 [file 41467_2018_7298_MOESM3_ESM.pdf]

```
#======================================================================

# CRYSTAL DATA

#----------------------------------------------------------------------

data_VESTA_phase_1


_chemical_name_common                  'large-pore phase'
_cell_length_a                         17.07860
_cell_length_b                         6.66300
_cell_length_c                         12.54517
_cell_angle_alpha                      89.99850
_cell_angle_beta                       89.99975
_cell_angle_gamma                      90.80737
_space_group_name_H-M_alt              'P 1'
_space_group_IT_number                 1

loop_
_space_group_symop_operation_xyz
   'x, y, z'

loop_
   _atom_site_label
   _atom_site_occupancy
   _atom_site_fract_x
   _atom_site_fract_y
   _atom_site_fract_z
   _atom_site_adp_type
   _atom_site_B_iso_or_equiv
   _atom_site_type_symbol
   O1         1.0     0.250793      0.001162      0.684456     Biso  1.000000 O
   O2         1.0     0.750856      0.501292      0.184462     Biso  1.000000 O
   O3         1.0     0.750799      0.001287      0.315549     Biso  1.000000 O
   O4         1.0     0.250863      0.501155      0.815542     Biso  1.000000 O
   O5         1.0     0.823858      0.840796      0.144196     Biso  1.000000 O
   O6         1.0     0.323760      0.340677      0.644040     Biso  1.000000 O
   O7         1.0     0.162404      0.822273      0.836619     Biso  1.000000 O
   O8         1.0     0.662309      0.322388      0.336428     Biso  1.000000 O
   O9         1.0     0.335981      0.178756      0.841738     Biso  1.000000 O
   O10        1.0     0.835901      0.678867      0.341899     Biso  1.000000 O
   O11        1.0     0.675799      0.158416      0.145044     Biso  1.000000 O
   O12        1.0     0.175698      0.658315      0.645197     Biso  1.000000 O
   O13        1.0     0.175795      0.158306      0.854934     Biso  1.000000 O
   O14        1.0     0.675712      0.658399      0.354844     Biso  1.000000 O
   O15        1.0     0.835981      0.178884      0.158237     Biso  1.000000 O
   O16        1.0     0.335891      0.678760      0.658104     Biso  1.000000 O
   O17        1.0     0.662391      0.822401      0.163438     Biso  1.000000 O
   O18        1.0     0.162309      0.322288      0.663553     Biso  1.000000 O
   O19        1.0     0.323869      0.840670      0.855815     Biso  1.000000 O
   O20        1.0     0.823761      0.340783      0.355942     Biso  1.000000 O
```

H1	1.0	0.273759	0.003808	0.614044	Biso	1.000000	H
H2	1.0	0.773864	0.503977	0.114075	Biso	1.000000	H
H3	1.0	0.773780	0.003968	0.385952	Biso	1.000000	H
H4	1.0	0.273914	0.503795	0.885902	Biso	1.000000	H
H5	1.0	0.922658	0.685880	0.030698	Biso	1.000000	H
H6	1.0	0.422336	0.185991	0.529983	Biso	1.000000	H
H7	1.0	0.045634	0.678718	0.920776	Biso	1.000000	H
H8	1.0	0.545313	0.178936	0.420074	Biso	1.000000	H
H9	1.0	0.454240	0.319936	0.922606	Biso	1.000000	H
H10	1.0	0.954140	0.819974	0.422835	Biso	1.000000	H
H11	1.0	0.577249	0.312625	0.032730	Biso	1.000000	H
H12	1.0	0.077149	0.812569	0.532956	Biso	1.000000	H
H13	1.0	0.077105	0.312711	0.966868	Biso	1.000000	H
H14	1.0	0.577152	0.812588	0.467112	Biso	1.000000	H
H15	1.0	0.954087	0.320141	0.076984	Biso	1.000000	H
H16	1.0	0.454137	0.819909	0.577223	Biso	1.000000	H
H17	1.0	0.545476	0.678920	0.079654	Biso	1.000000	H
H18	1.0	0.045311	0.178879	0.579868	Biso	1.000000	H
H19	1.0	0.422506	0.685966	0.969731	Biso	1.000000	H
H20	1.0	0.922340	0.186019	0.469950	Biso	1.000000	H
Al1	1.0	0.249323	0.750512	0.749206	Biso	1.000000	Al
Al2	1.0	0.749322	0.250631	0.249190	Biso	1.000000	Al
Al3	1.0	0.749324	0.750630	0.250818	Biso	1.000000	Al
Al4	1.0	0.249321	0.250514	0.750793	Biso	1.000000	Al
C1	1.0	0.956853	0.822316	0.016122	Biso	1.000000	C
C2	1.0	0.456611	0.322380	0.515622	Biso	1.000000	C
C3	1.0	0.024955	0.818047	0.955604	Biso	1.000000	C
C4	1.0	0.524716	0.318168	0.455105	Biso	1.000000	C
C5	1.0	0.474541	0.180663	0.957893	Biso	1.000000	C
C6	1.0	0.974425	0.680684	0.458121	Biso	1.000000	C
C7	1.0	0.542668	0.176661	0.018372	Biso	1.000000	C
C8	1.0	0.042551	0.676631	0.518599	Biso	1.000000	C
C9	1.0	0.042586	0.176713	0.981408	Biso	1.000000	C
C10	1.0	0.542553	0.676640	0.481439	Biso	1.000000	C
C11	1.0	0.974457	0.180776	0.041881	Biso	1.000000	C
C12	1.0	0.474424	0.680647	0.541911	Biso	1.000000	C
C13	1.0	0.524867	0.818159	0.044642	Biso	1.000000	C
C14	1.0	0.024715	0.318138	0.544862	Biso	1.000000	C
C15	1.0	0.456766	0.822366	0.984118	Biso	1.000000	C
C16	1.0	0.956614	0.322397	0.484341	Biso	1.000000	C
C17	1.0	0.931044	0.003673	0.059484	Biso	1.000000	C
C18	1.0	0.430912	0.503610	0.559260	Biso	1.000000	C
C19	1.0	0.068173	0.995311	0.937913	Biso	1.000000	C
C20	1.0	0.568043	0.495359	0.437690	Biso	1.000000	C
C21	1.0	0.431045	0.003611	0.940519	Biso	1.000000	C
C22	1.0	0.930915	0.503660	0.440736	Biso	1.000000	C
C23	1.0	0.568169	0.995371	0.062102	Biso	1.000000	C
C24	1.0	0.068040	0.495317	0.562315	Biso	1.000000	C
C25	1.0	0.858271	0.008053	0.125474	Biso	1.000000	C
C26	1.0	0.358164	0.507942	0.625301	Biso	1.000000	C
C27	1.0	0.140739	0.991605	0.871608	Biso	1.000000	C
C28	1.0	0.640636	0.491695	0.371440	Biso	1.000000	C

C29	1.0	0.358275	0.007941	0.874522	Biso	1.000000 C
C30	1.0	0.858170	0.508034	0.374692	Biso	1.000000 C
C31	1.0	0.640735	0.991712	0.128409	Biso	1.000000 C
C32	1.0	0.140630	0.491614	0.628570	Biso	1.000000 C
