# Supplementary material for: Tuning the balance between dispersion and entropy to design temperature-responsive flexible metal-organic frameworks
Source: Nat Commun. 2018 Nov 21;9:4899. doi: 10.1038/s41467-018-07298-4 (PMC6249296; doi:10.1038/s41467-018-07298-4)
Supplement: Supplementary file 4 — Supplementary Data 2 [file 41467_2018_7298_MOESM4_ESM.pdf]

```
#======================================================================

# CRYSTAL DATA

#----------------------------------------------------------------------

data_VESTA_phase_1


_chemical_name_common                  'narrow-pore phase'
_cell_length_a                         19.24632
_cell_length_b                         6.66078
_cell_length_c                         6.76503
_cell_angle_alpha                      90
_cell_angle_beta                       90
_cell_angle_gamma                      95.01920
_space_group_name_H-M_alt              'P 1'
_space_group_IT_number                 1

loop_
_space_group_symop_operation_xyz
   'x, y, z'

loop_
   _atom_site_label
   _atom_site_occupancy
   _atom_site_fract_x
   _atom_site_fract_y
   _atom_site_fract_z
   _atom_site_adp_type
   _atom_site_B_iso_or_equiv
   _atom_site_type_symbol
   Al1        1.0     0.500000     0.500000     0.000000    Biso  1.000000 Al
   Al2        1.0     0.000000     0.000000     0.500000    Biso  1.000000 Al
   Al3        1.0     0.500000     0.000000     0.000000    Biso  1.000000 Al
   Al4        1.0     0.000000     0.500000     0.500000    Biso  1.000000 Al
   O1         1.0     0.000000     0.250000     0.377291    Biso  1.000000 O
   O2         1.0     0.500000     0.750000     0.877291    Biso  1.000000 O
   O3         1.0     0.000000     0.750000     0.622709    Biso  1.000000 O
   O4         1.0     0.500000     0.250000     0.122709    Biso  1.000000 O
   O5         1.0     0.063021     0.612114     0.301957    Biso  1.000000 O
   O6         1.0     0.563021     0.112114     0.801957    Biso  1.000000 O
   O7         1.0     0.936979     0.887886     0.301957    Biso  1.000000 O
   O8         1.0     0.436979     0.387886     0.801957    Biso  1.000000 O
   O9         1.0     0.936979     0.387886     0.698043    Biso  1.000000 O
   O10        1.0     0.436979     0.887886     0.198043    Biso  1.000000 O
   O11        1.0     0.063021     0.112114     0.698043    Biso  1.000000 O
   O12        1.0     0.563021     0.612114     0.198043    Biso  1.000000 O
   O13        1.0     0.082026     0.947460     0.349814    Biso  1.000000 O
   O14        1.0     0.582026     0.447460     0.849813    Biso  1.000000 O
   O15        1.0     0.917974     0.552540     0.349813    Biso  1.000000 O
   O16        1.0     0.417974     0.052540     0.849814    Biso  1.000000 O
```

O17	1.0	0.917974	0.052540	0.650186	Biso	1.000000	O
O18	1.0	0.417975	0.552540	0.150187	Biso	1.000000	O
O19	1.0	0.082026	0.447460	0.650187	Biso	1.000000	O
O20	1.0	0.582026	0.947460	0.150186	Biso	1.000000	O
H1	1.0	0.000000	0.250000	0.234446	Biso	1.000000	H
H2	1.0	0.500000	0.750000	0.734446	Biso	1.000000	H
H3	1.0	0.000000	0.750000	0.765554	Biso	1.000000	H
H4	1.0	0.500000	0.250000	0.265554	Biso	1.000000	H
H5	1.0	0.205003	0.079500	0.328144	Biso	1.000000	H
H6	1.0	0.705003	0.579500	0.828144	Biso	1.000000	H
H7	1.0	0.794997	0.420500	0.328144	Biso	1.000000	H
H8	1.0	0.294997	0.920500	0.828145	Biso	1.000000	H
H9	1.0	0.794997	0.920500	0.671856	Biso	1.000000	H
H10	1.0	0.294997	0.420500	0.171856	Biso	1.000000	H
H11	1.0	0.205003	0.579500	0.671856	Biso	1.000000	H
H12	1.0	0.705003	0.079500	0.171856	Biso	1.000000	H
H13	1.0	0.333655	0.053099	0.293517	Biso	1.000000	H
H14	1.0	0.833655	0.553099	0.793517	Biso	1.000000	H
H15	1.0	0.666345	0.446901	0.293517	Biso	1.000000	H
H16	1.0	0.166345	0.946901	0.793517	Biso	1.000000	H
H17	1.0	0.666345	0.946901	0.706483	Biso	1.000000	H
H18	1.0	0.166345	0.446901	0.206483	Biso	1.000000	H
H19	1.0	0.333655	0.553099	0.706483	Biso	1.000000	H
H20	1.0	0.833655	0.053099	0.206483	Biso	1.000000	H
C1	1.0	0.178281	0.765109	0.267360	Biso	1.000000	C
C2	1.0	0.678281	0.265109	0.767360	Biso	1.000000	C
C3	1.0	0.821719	0.734891	0.267360	Biso	1.000000	C
C4	1.0	0.321719	0.234891	0.767360	Biso	1.000000	C
C5	1.0	0.821719	0.234891	0.732640	Biso	1.000000	C
C6	1.0	0.321719	0.734891	0.232640	Biso	1.000000	C
C7	1.0	0.178281	0.265109	0.732640	Biso	1.000000	C
C8	1.0	0.678281	0.765109	0.232640	Biso	1.000000	C
C9	1.0	0.225260	0.936249	0.292907	Biso	1.000000	C
C10	1.0	0.725260	0.436249	0.792907	Biso	1.000000	C
C11	1.0	0.774740	0.563751	0.292907	Biso	1.000000	C
C12	1.0	0.274740	0.063751	0.792907	Biso	1.000000	C
C13	1.0	0.774740	0.063751	0.707093	Biso	1.000000	C
C14	1.0	0.274740	0.563751	0.207093	Biso	1.000000	C
C15	1.0	0.225260	0.436249	0.707093	Biso	1.000000	C
C16	1.0	0.725260	0.936249	0.207093	Biso	1.000000	C
C17	1.0	0.102396	0.776407	0.306224	Biso	1.000000	C
C18	1.0	0.602396	0.276407	0.806224	Biso	1.000000	C
C19	1.0	0.897604	0.723593	0.306224	Biso	1.000000	C
C20	1.0	0.397604	0.223593	0.806224	Biso	1.000000	C
C21	1.0	0.897604	0.223593	0.693776	Biso	1.000000	C
C22	1.0	0.397604	0.723593	0.193776	Biso	1.000000	C
C23	1.0	0.102396	0.276407	0.693776	Biso	1.000000	C
C24	1.0	0.602396	0.776407	0.193776	Biso	1.000000	C
C25	1.0	0.296537	0.921234	0.275902	Biso	1.000000	C
C26	1.0	0.796537	0.421234	0.775901	Biso	1.000000	C
C27	1.0	0.703463	0.578766	0.275901	Biso	1.000000	C
C28	1.0	0.203463	0.078766	0.775902	Biso	1.000000	C

C29	1.0	0.703463	0.078766	0.724098	Biso	1.000000	C
C30	1.0	0.203463	0.578766	0.224099	Biso	1.000000	C
C31	1.0	0.296537	0.421234	0.724099	Biso	1.000000	C
C32	1.0	0.796537	0.921234	0.224098	Biso	1.000000	C
